# Unravelling Catalytic Divergence in Mo- and Fe-Only Nitrogenases: The Role of the Heterometal-Site and Protein Environment from QM/MM Insights

**DOI:** 10.1021/jacs.5c20796

**Published:** 2025-12-16

**Authors:** Justin P. Joyce, Ragnar Bjornsson, Serena DeBeer

**Affiliations:** † 28313 Max Planck Institute for Chemical Energy Conversion, 45470 Mülheim an der Ruhr, Germany; ‡ 27051 Univ. Grenoble Alpes, CNRS, CEA, LCBM (UMR 5249), F-38000 Grenoble, France

## Abstract

Two key characteristics differentiate molybdenum-dependent (MoFe) and iron-only (FeFe) nitrogenases: their efficacy for N_2_ fixation and their product distributions for CO_2_ reduction, yielding HCO_2_^–^ and CH_4_, respectively. Despite their divergent properties, prior research argues that the distinct cofactors share a common mechanism and equivalent structures with the addition of “*n*” electrons and protons at their E*_n_* catalytic states. The proposed equivalence between the cofactors was foundational in the assignment of an Fe-hydride at their E_1_ states based on interpretation of FeFeco’s photolabile and thermolabile E_1_ isomers (*Inorg. Chem*. **2022**, 61, 5459–5464). However, the E_0_ state crystal structures of FeMoco and FeFeco have sharp, previously unaddressed distinctions in their metal–metal distances and proximal residue identities at their respective octahedral M-sites (M = Mo or Fe). Herein, we study QM/MM models of the E_0_ and E_1_ states of FeMoco and FeFeco to distinguish their geometric and electronic structures. Our analysis shows diminished metal–metal bonding and increased hydrogen bonding at FeFeco’s M-site, supporting the presence of two energetically low-lying E_1_ states differentiated by protonation of a μ_3_- or μ_2_-sulfide. The calculated thermodynamic and kinetic properties of FeFeco’s sulfide-protonated states agree with experimental data, without invoking Fe-hydride formation. Unlike FeFeco, FeMoco’s E_1_ state strictly favors μ_2_-sulfide protonation. FeFeco’s distinct μ_3_-sulfide-protonated E_1_ isomer has a five-coordinate, reduced M-site that could explain its divergent reactivity relative to FeMoco.

## Graphical abstract

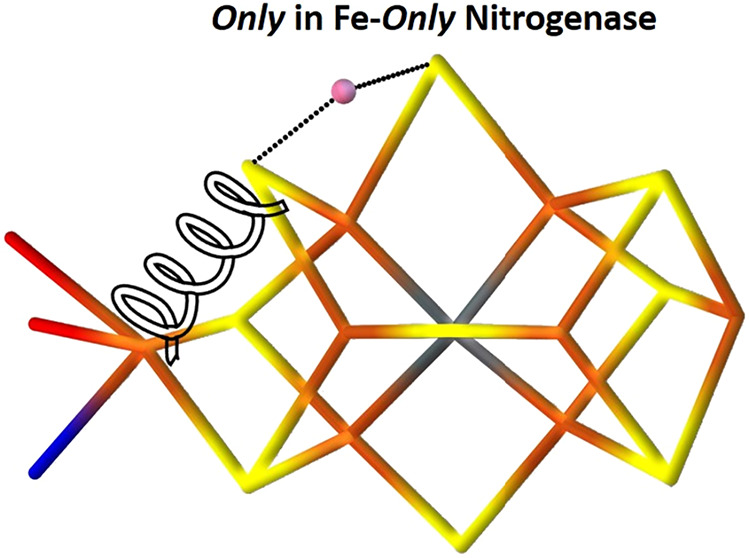

## Introduction

Nitrogenases can be divided into three main classes: Mo-dependent, V-dependent, and Fe-only forms, which are distinguished by the homocitrate-bound metal in the Fe­(M)­co cluster of their cofactors (M = Mo, V, or Fe). These cofactors, commonly referred to as FeMoco, FeVco, and FeFeco, are the active sites for substrate binding and reduction within the catalytic proteins, referred to as MoFe, VFe, and FeFe, respectively. The catalytic (M)Fe protein also contains an [Fe_8_S_7_] P-cluster that transfers electrons to each E*_n_* state of Fe­(M)­co during the Lowe-Thorneley cycle, where “*n*” denotes the number of electrons (e^–^) and protons (H^+^) added to the system.^1–4^ While named for their ability to reduce atmospheric nitrogen (N_2_) to bioavailable ammonia (NH_3_), nitrogenases can facilitate reduction of a diverse range of substrates that include protons, acetylene (C_2_H_2_), carbon monoxide (CO), and carbon dioxide (CO_2_).^5–7^

VFe and FeFe are typically referred to as “alternative” nitrogenases because MoFe is the most efficient nitrogenase for N_2_ reduction.^8–10^ However, the remarkable catalytic ability of the underexplored class of “alternative” nitrogenases has been increasingly highlighted since 2010. Representative VFe can catalyze the reductive C–C bond coupling of CO, producing ethylene (C_2_H_4_) that is analogous to the Fischer–Tropsch process.^11,12^ While exciting, the foundational properties of VFe, including its cofactor’s E_0_ oxidation and spin state, are unsettled.^13,14^ More recently, FeFe has demonstrated the singular ability to fully reduce CO_2_ to methane (CH_4_), a biological analogue to the Sabatier process.^15–18^ The catalytic properties of FeFe also include the full reduction of C_2_H_2_ to ethane (C_2_H_6_) and higher H_2_ production activity.^19^ Comparatively, the sole products for the reduction of CO_2_ and C_2_H_2_ in MoFe are formate (HCO_2_^–^) and C_2_H_4_, respectively. Currently, there is no consensus on the cause of the divergent reactivities between FeMoco and FeFeco.

The crystal structure of FeFeco has only recently been reported by Einsle and co-workers.^20^ A combination of crystallography and valence-to-core X-ray emission spectroscopy has confirmed that all three Fe­(M)­co clusters have an interstitial carbide that fuses two Fe–S clusters designated as the homo-[Fe_4_S_3_C] and heterocubane [(homocitrate)­MFe_3_S_3_C].^21–25^ A structural schematic of the FeMoco and FeFeco E_0_ states highlights their similar cofactor structures, which has generally led to the assumption that their E*_n_* states are equivalent (Figure 1). We have proposed, and others supported, a model for the μ_2_S2B protonation in FeMoco’s E_1_ state, from complementary X-ray spectroscopic and QM/MM data.^26,27^ In a recent QM/MM study, Ryde assigned an analogous μ_2_S2B protonated E_1_ ground state for FeFeco.^28,29^ However, Hoffman and co-workers interpreted FeFeco’s photolabile and thermolabile E_1_ state’s EPR spectrum as Fe-hydrides and inferred that this also applies to FeMoco’s E_1_ state.^30,31^ Similar to their assignment of FeMoco’s E_2_ and E_4_ states, their interpretation was rooted in the photochemical reductive elimination of H_2_ demonstrated in metal dihydrides.^32–34^ However, complementary ^1,2^H ENDOR spectroscopy has not been reported to unambiguously assign the analogous photolability of FeFeco’s proposed monohydride E_1_ state.^35^ Supporting their interpretation, FeFeco’s E_1_ photoconversion at 450 nm shows a kinetic isotope effect (KIE) of approximately 2.4, which is comparable to the KIEs of H_2_ production in FeMoco’s E_2_ state.^36^

**Figure 1:**
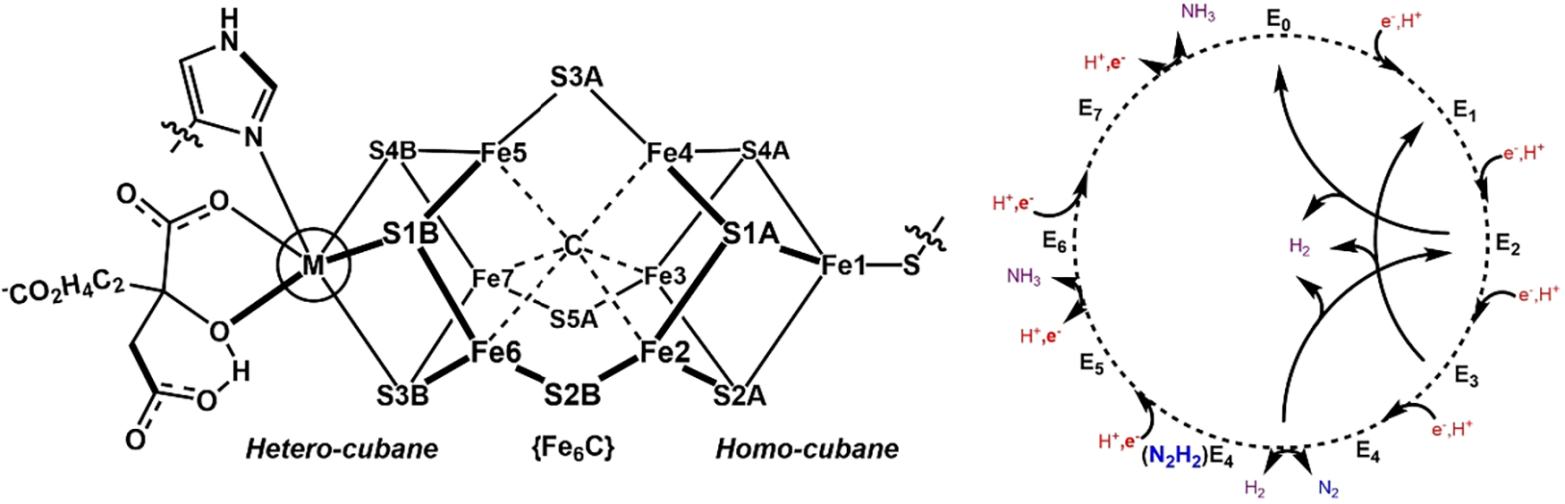
(*left*) The structure of the nitrogenase cofactors Fe­(M)­co, where M = Mo or Fe. The Fe- and S atoms are labeled with respect to their crystallographic notation. The sections of the cofactor are labeled. (*right*) The Loewe-Thorneley kinetic cycle for nitrogenase’s reduction of nitrogen to ammonia. The addition of one electron and proton to each proceeding E*_n_* state is shown.

A remarkable aspect of FeFeco’s crystal structure unaddressed by Einsle and co-workers is the marked elongation of its average M-Fe distances by 0.19 Å compared with FeMoco.^20^ The cofactor’s Fe K-edge Extended X-ray Absorption Fine Structure (EXAFS) spectroscopy similarly indicates a 0.23 Å expansion of FeFeco’s M-Fe distances.^27,37^ Also notable are the unique lysine residues (Lys83, Lys339) in FeFeco whose ammonium groups exhibit hydrogen bonding with the μ_3_-sulfides at the M-site’s primary coordination sphere. The same noncovalent interactions are not exhibited by FeMoco’s analogous arginine residues (Arg96, Arg359). While lysine and arginine are both cationic residues of comparable size, lysine’s ammonium group (p*K*_a_ = 10.4) is more acidic than arginine’s resonance-stabilized guanidinium group (p*K*_a_ = 13.8).^38^ The respective Arg96 and Lys83 residues of FeMoco and FeFeco are near the μ_3_S3B center that Dance’s computational studies have assigned as the cofactor’s initial protonation site.^39–42^

The substitution reactions at Fe–S clusters are known to be acid-catalyzed, with their protonation rate approximately 4 orders of magnitude slower than the diffusion-controlled limit.^43,44^ The kinetics for Fe–S cluster protonation are attributed to the disruption of the cubane architecture, with dissociation of an Fe–S bond accompanying the protonation of the μ_3_-sulfide site.^45,46^ ^57^Fe-labeling studies from Suess have similarly illustrated the fluxional cubane framework in Fe–S clusters.^47^ Reported by Henderson, the protonation rate of Fe–S clusters by ammonium-based acids decreases up to 100-fold in Mo-containing systems, and this sluggish protonation is interpreted to support N_2_ binding while suppressing H_2_ production.^48,49^

Herein, we perform a QM/MM study comparing the electronic structures of E_0_ and E_1_ of FeMoco and FeFeco. FeFeco’s M-site notably does not exhibit the covalent metal–metal bonding with adjacent Fe-sites that we have previously detailed in FeMoco. We report that the energetically favorable E_1_ structure for FeMoco and FeFeco has a protonated μ_2_-sulfide (μ_2_S2B) and a reduced heterocubane. However, FeFeco has a low-lying E_1_ isomer (Δ*E* = +3.5 kcal mol^–1^) where a five-coordinate, reduced M-site accompanies μ_3_-sulfide (μ_3_S3B) protonation, proximal to the cofactor’s acidic lysines. Our calculated properties, for FeFeco’s E_1_ isomers, are consistent with their experimentally reported photolytic properties, including the kinetic isotope effect, and are consistent with sulfide-protonated, rather than Fe-hydride, structures. The coordinatively unsaturated and reduced M-site in FeFeco’s distinct E_1_ isomer could provide future insight into its recently highlighted reduction of CO_2_, as well as its greater H_2_ production accompanying N_2_ reduction.

## Methodology

Our QM/MM model of FeFeco is based on the recent crystal structure reported by Einsle (PDB: 8BOQ).^20^ The crystallographic model for 8POQ represents the disorder at the μ_2_S2B site as equal occupancy of a sulfide and a hydroxide. The model similarly represents the disorder of the Gln176 residue’s conformation. FeFeco’s resting state is consistent with the μ_2_S2B identity and Gln176 hydrogen bonding to the homocitrate ligand. FeFeco’s crystal structure has respective resolutions of 1.6, 2.6, and 2.3 Å along the *a*, *b*, and *c* crystallographic axes. The structure of FeFeco was solved by geometric constraints from a FeMoco-based model. Our QM/MM model for FeMoco is based on the 1.0 Å resolved crystal structure (PDB 3U7Q) that has been previously detailed.^22,50^

The protonation states of titratable residues were assigned from the inspection of the structure’s hydrogen bonding network. The Arg, Lys, Asp, and Glu residues were all assigned as charged. The cysteines coordinated to Fe-sites were assigned as deprotonated (α-49^Cys^, α-75^Cys^, α-138^Cys^, α-257^Cys^, β-20^Cys^, β-45^Cys^, β-104^Cys^). The histidine coordinated to FeFeco’s M-site is modeled as its δ-protonation state. The His180 residue is assigned the ε-protonation state that hydrogen bonds to the μ_2_S2B site. The listed histidine residues were modeled by their δ-protonation state (α-3^His^, α-18^His^, α-70^His^, α-203^His^, α-342^His^, α-345^His^, α-364^His^, α-426^His^, α-452^His^, β-40^His^, β-56^His^, β-69^His^, β-140^His^, β-162^His^, β-221^His^, β-294^His^, β-308^His^, β-451^His^, γ-29^His^, γ-79^His^, γ-119^His^). The remaining histidine residues were assigned to their ε-protonation state. The homocitrate ligand’s oxygen-containing functional groups were assigned as one hydroxyl and three carboxylate groups that is a 3- charge.^29^

The QM/MM program ASH was used,^51^ providing a flexible interface to the OpenMM molecular mechanics library^52^ and the ORCA quantum chemistry code (ORCA v5.03).^53,54^ For FeFe, an MM model of the solvated protein was first prepared based on the CHARMM36 force field.^55^ Lennard-Jones parameters for Zn^2+^ were used at the Fe-sites. The [Fe_8_S_9_C]^2–^ atomic charges (used only during MM simulations) were derived from the Hirshfeld population analysis of the cluster with respect to the density functional and basis set detailed below. We assigned the P-clusters as its fully reduced (P^N^) state, applying the same force field parameters from our previous study.^50^ The homocitrate force field parameters were also taken from our previous studies. The protein was solvated in a cubic box with lengths of 14.3536 nm, with water molecules modeled as TIP3P.^56^ The protein was assigned a neutral charge, and Na^+^ and Cl^–^ anions were added to give an ionic strength of 0.1 M. This gave a solvated protein model size of 296,836 atoms. The system was next equilibrated within a canonical (NVT) ensemble with all Fe–S clusters frozen. The MD simulations were performed for 5.0 ns at a temperature of 300 K, using a Langevin Middle Integrator and a 1 ps^–1^ coupling frequency. A nonbonded cutoff of 12 Å was used during these simulations.

QM/MM calculations were performed for both MoFe and FeFe proteins, where the covalent QM/MM boundary was treated with link atoms and charge-shifting.^57^ Geometries were optimized with the geomeTRIC optimization library^58^ using HDLC coordinates^59^ of an active region, defined as all residues within a 10 Å radius from the interstitial carbide (959 atoms). The QM-region was selected to include the primary and secondary coordination spheres of FeMoco and FeFeco’s respective [MoFe_7_S_9_C] and [Fe_8_S_9_C] clusters. The secondary coordination sphere is defined as residue side chains whose heavy atom separation from the metallocluster was less than the sum of their respective van der Waals radii.^60^ A crystallographic water molecule is included for both cofactors’ QM-region that exhibits a hydrogen bond to the homocitrate ligand’s coordinated carboxylate group. Assigned below, the residues define a QM-region of 168 and 163 when including the link atoms with respect to FeMoco and FeFeco’s E_0_ state. The QM active regions of FeMoco and FeFeco’s E_0_ state are shown in Figures S1 and S2, respectively.

FeMoco QM-region: Val70, Arg96, Gln191, His195, Cys275, Ser278, Gly356, Gly357, Arg359, Glu380, Phe381, His442, and HOH519

FeFeco QM-region: Val57, Lys83, Gln176, His180, Cys257, Ser260, Gly337, Ser338, Lys339, Lys361, Phe362, His423, and HOH887

The r^2^SCAN density functional^61^ was utilized in combination with the D4 dispersion correction,^62,63^ and the ZORA scalar relativistic Hamiltonian,^64,65^ a protocol that has been benchmarked for the geometric and electronic structure of spin-coupled iron–sulfur based systems.^66^ The relativistically recontracted ZORA-def2-TZVP basis set was used for the Fe and S centers and the interstitial carbide of the cofactors.^67,68^ The E_1_ state’s additional hydrogen group also used the ZORA-def2-TZVP basis set. The all-electron SARC-ZORA-TZVP basis set was used for Mo.^69^ All of the remaining atoms used the smaller ZORA-def2-SVP basis set. The Split-RI-J approximation in ORCA was used together with a decontracted auxiliary basis set (“SARC/J”).^70,71^ The cofactors’ relative E_1_ isomer energies were also characterized at the same level of theory by the TPSSh^72^ density functional.

The FeMoco and FeFeco E_0_ and E_1_ states were calculated with broken-symmetry-density functional theory (BS-DFT) within the QM/MM model. The total charge of FeMoco and FeFeco’s E_0_ state was respectively assigned as [MoFe_7_S_9_C]^1–^ and [Fe_8_S_9_C]^2–^. The ferromagnetic solutions for local high-spin metal centers of [Mo^3+^Fe_4_^3+^Fe_3_^2+^] and [Fe_4_^3+^Fe_4_^2+^] are respectively *M*_s_ = 35/2 and 18. The BS-solutions were found by inverting the net spin on specific metal sites from α- to β-spin, converging to *M*_s_ = 3/2 and 0 states with respect to FeMoco and FeFeco. The respective E_1_ states of FeMoco and FeFeco were assigned *M*_s_ = 2 and 1/2 states. Both FeMoco and FeFeco have eight open-shell metal centers with dominant α-(n_α_) or β-(n_β_) spin densities. eq 1 assigns 70 possible broken-symmetry solutions (n_α,β_), assuming an equal number of n_α_ and n_β_ spin centers. This manuscript’s BS-solution notation specifies the metal sites with n_β_ spin with respect to the crystallographic notation of the nitrogenase cofactors shown in Figure 1. Previous studies on FeMoco have used a BS-notation that assigns only Fe-centers with n_β_ spin, lowering the number of possible solutions to thirty-five, because of the minimal spin density localized on the Mo-center.^28,50^ Both formalisms are usually related to Noodleman’s original notation, the cofactor’s approximate *C*_3_-symmetry having ten distinct BS-solutions.^73^ The connections between the three notation formalisms are provided in Table 1.

1 $$n_{\alpha ,\beta }=\sum \frac{\left(n_{\alpha }+n_{\beta }\right)!}{\left(n_{\alpha }!{)(n}_{\beta }!\right)}$$

**Table 1:** Relationship between the Notations Used to Specify the BS-Solution in the Nitrogenase Cofactor

Noodleman Notation^73^ (*n*_α_ = 3, *n*_β_ = 2)	Previous Notation^50^ (*n*_α_ = 4, *n*_β_ = 3)	Current Notation (*n*_α_ = 4, *n*_β_ = 4)
BS1	BS-567	BS-567M, BS-1234
BS2	BS-234	BS-234M, BS-1567
BS3	BS-123	BS-123M, BS-4567
	BS-124	BS-124M, BS-3567
	BS-134	BS-134M, BS-2567
BS4	BS-257	BS-257M, BS-1346
	BS-356	BS-356M, BS-1247
	BS-467	BS-467M, BS-1235
BS5	BS-256	BS-256M, BS-1347
	BS-357	BS-357M, BS-1246
	BS-367	BS-367M, BS-1245
	BS-456	BS-456M, BS-1237
	BS-457	BS-457M, BS-1236
	BS-267	BS-267M, BS-1345
BS6	BS-156	BS-156M, BS-2347
	BS-157	BS-157M, BS-2346
	BS-167	BS-167M, BS-2345
BS7	BS-235	BS-235M, BS-1467
	BS-247	BS-247M, BS-1356
	BS-346	BS-346M, BS-1257
BS8	BS-245	BS-245M, BS-1367
	BS-345	BS-345M, BS-1267
	BS-236	BS-236M, BS-1457
	BS-246	BS-246M, BS-1357
	BS-237	BS-237M, BS-1456
	BS-347	BS-347M, BS-1256
BS9	BS-126	BS-126M, BS-3457
	BS-137	BS-137M, BS-2456
	BS-145	BS-145M, BS-2367
BS10	BS-127	BS-127M, BS-3456
	BS-136	BS-136M, BS-2457
	BS-135	BS-135M, BS-2467
	BS-147	BS-147M, BS-2356
	BS-125	BS-125M, BS-3467
	BS-146	BS-146M, BS-2357

The cofactors’ BS7 solutions are usually the most stable, specifically the BS-235M, −247M, and −346M configurations in the current notation. The perturbation of a μ_2_-sulfide can stabilize a BS10 solution, showing a parallel spin arrangement of the bridged Fe-sites (Figure S3).^74,75^ The most stable BS-solution for the cofactors’ E_0_ state and E_1_ isomers was selected from their optimized BS-235M, −247M, and −346M solutions (Tables S1–S3). BS-solutions, belonging to the BS10 class for the μ_2_S2B and μ_2_S5A isomers, were also considered. All possible BS-solutions were explored for FeFeco’s μ_3_S3B isomer (Table S4). The spin populations of select E_0_ and E_1_ isomers’ metal sites were calculated by Hirshfeld Population analysis, provided in Section S3.^76^

The metal center oxidation and spin states and their magnetic interactions are analyzed via Pipek-Mezey (PM) localized orbitals.^77^ The PM-localized orbitals maximize the atom-centered Mulliken charge population, highlighting discrete bonding interactions possessing distinct α-(φ_α_) and β-spin (φ_β_) in a broken symmetry framework. The associated spin densities from the PM-localized orbitals (ρ_α,β_) are defined with eqs 2, with respect to the total of α-(x_α_) and β-(x_β_) electrons in a two-center bonding interaction between sites “A” and “B”.

2 $$\rho_{\alpha ,\beta }\left(A,B\right)=\sum_{i=1}^{x_{\alpha }}\phi_{\alpha ,i}^{2}(A,B)-\sum_{i=1}^{x_{\beta }}\phi_{\beta ,i}^{2}\left(A,B\right)=\rho_{\alpha }(A,B)-\rho_{\beta }\left(A,B\right)$$

The Extended Transition State-Natural Orbitals for Chemical Valence (ETS-NOCV) method^78^ was performed by the Multiwfn v3.8 electronic structure program.^79,80^ The method was used for the BS-235M solution of FeMoco and FeFeco’s E_0_ state with respect to discrete residues. The fragments were assigned to a select residue (“A”) and the remaining atoms in the QM-region (“B”). The charges, multiplicities, and BS-solution of the fragments were selected to be consistent with the total system (“AB”). The NOCV pair densities and values are the sum of the α- and β-spin components.

The minimum energy path (MEP) between E_1_ isomers was obtained from the Nudged Elastic Band-Climbing Image (NEB-CI) method as implemented in ASH.^81–83^ The minima for the reactants and products were independently optimized. The same BS-solution was used for the products, reactants, and intermediate images. The saddle point geometry found by the NEB-CI method was independently optimized, and Hessian calculations were performed to confirm all minima and saddle points and to calculate free energy corrections. The vibrational frequencies were calculated by using a numerical one-point formula partial Hessian approach. The room temperature kinetic isotope effects (KIE) were calculated by the change in the reactant and transition state’s zero-point energy (ZPE) when the E_1_ state has an additional hydrogen or deuterium group.

## Results and Discussion

### Resting State (E_0_) Properties

In FeFeco, we assign three near-degenerate (∼1 kcal mol^–1^) E_0_ ground states, labeled as BS-235M, −247M, and −346M, and confirm analogous states in FeMoco’s, consistent with previous reports (Table S1).^29,50^ The properties of FeMoco’s E_0_ state, by spatially resolved anomalous dispersion (SpReAD) refinement, single-crystal EPR, ^13^C ENDOR and ^57^Fe Mössbauer spectroscopies independently support the electronic structure of its BS-235M, −247M and −346M solutions.^84–87^ While near-degenerate, the cofactor’s descent from *C*_3_-symmetry results in distinct geometric and electronic structures between BS-solutions, with BS-235M best reproducing the trends in metal–metal distances of the X-ray structure (Tables S6–S7).

We compare the metal–metal distances for the BS-235M QM/MM optimized E_0_ structures of FeMoco and FeFeco (Figure 2), which are consistent with the distinct structural features suggested by both X-ray crystallography and Fe K-edge EXAFS of MoFe and FeFe proteins.^20,22,27,37^ The expansion of FeFeco’s M-Fe distances varies between Fe-sites, increasing in the following order: M-Fe5 (+0.07 Å), M-Fe7 (+0.22 Å), and M-Fe6 (+0.29 Å), which increases the volume of the MFe_3_ tetrahedron of the heterocubane from 2.15 to 2.42 Å^3^ (+12.6%).^88^ For context, the crystallographic characterization of the full [Fe_4_S_4_]^4+,0^ redox series has recently assigned a change of 2.45 to 2.29 Å^3^ (−6.5%) in the Fe_4_ volume between its fully oxidized and fully reduced states.^89^ The heterocubane’s corresponding metal–sulfur bond lengths, previously characterized by the bond valence method, exhibit minor differentiation between FeMoco and FeFeco, suggesting the M-site structural perturbations do not result from changes in metal–sulfur covalency.^90^ We do not report significant deviation between the cofactor’s Fe_4_ and S_3_C tetrahedron volumes, which are reported in Table S8. The covalent radii are used to calculate the formal shortness ratio, treating all metal–metal distances equivalently.^91^ The covalent radii of six-coordinate Mo (1.54 Å) and high-spin Fe (1.52 Å) are near-equivalent, and fluctuate by ∼0.01 Å between their Fe^2+/3+^ and Mo^3+/4+^ oxidation states for octahedral complexes, suggesting a size difference is not responsible for their distinct distances.^92^

**Figure 2:**
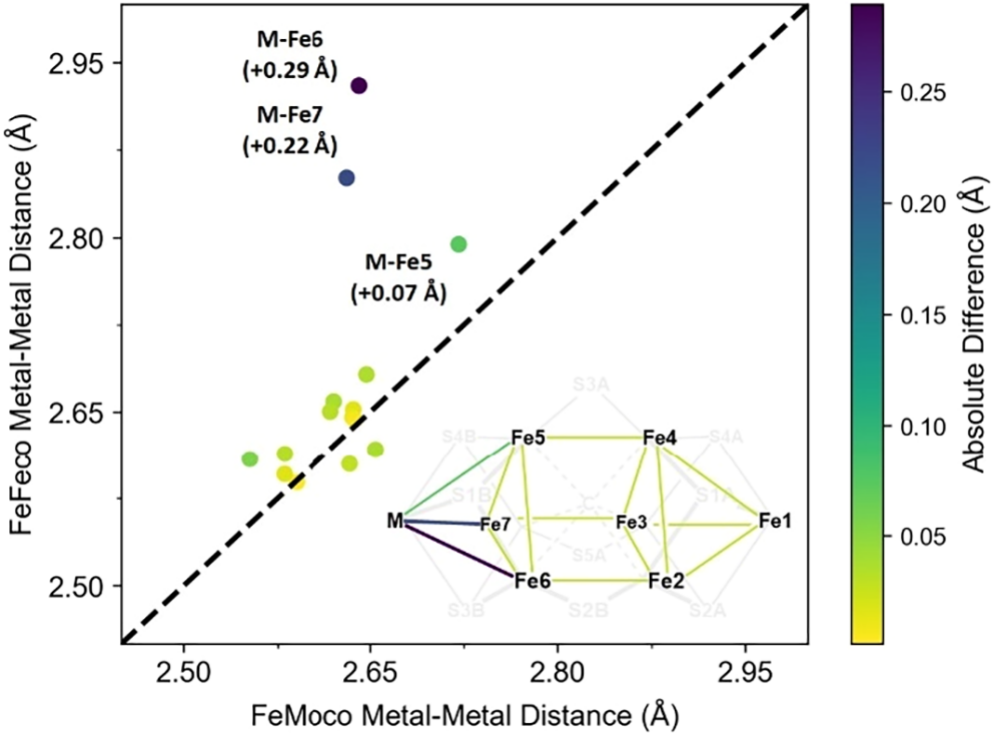
BS-235M QM/MM optimized metal–metal distances of FeMoco (*x*-axis) and FeFeco’s (*y*-axis) E_0_ state. The color for the metal–metal bonds (*inset*) and data points corresponds to the absolute difference between the cofactors’ analogous distances (color bar, *right*). The dashed black line, representing no deviation between cofactors, provides a guide for the eye. The expansion of FeFeco’s M-Fe bonds is highlighted for M-Fe5 (green), M-Fe6 (purple), and M-Fe7 (blue).

The M-site’s octahedral *t*_2g_ orbitals are directed toward the heterocubane’s three Fe-sites, where the direct overlap of their d-orbitals introduces metal–metal bonding, which can exhibit diverse electronic structures characterized as ferro- or antiferromagnetic or covalent.^91^ We characterize the M-site’s localized orbitals, using the ρ_α,β_(M,Fe) spin density defined in eq 2 to assign the electronic structure origin of the cofactor’s disparate M-Fe distances, with blue and green indicating α- and β-spin, respectively (Figure 3). The corresponding localized orbitals (Figures S5 and S6) demonstrate how our ρ_α,β_(A,B) plots consolidate multiple orbitals into a single isosurface, while highlighting their distinct spins.

**Figure 3:**
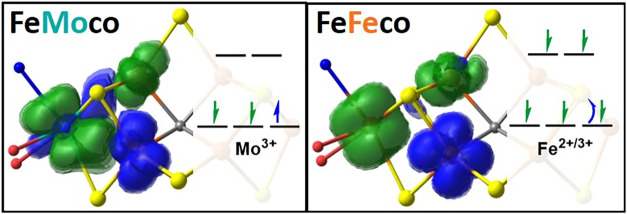
Pipek-Mezey localized spin densities for the M-Fe bonding in FeMoco (*left*) and FeFeco’s (*right*) E_0_ (BS-235M) state. Blue and green, respectively, assign spin densities of α-(up) and β-(down) character. The M-site’s local electronic structure is included with the inset orbital diagram, the curved arrow assigning the mixed-valence state’s delocalized electron.

The Mo-site exhibits spin-polarized bonds to the adjacent Fe-sites, with an inequal distribution of α and β electrons. Such spin-polarized bonds are analogous to the unequal distribution of charge in traditional polar-covalent bonds and have been observed in open-shell systems.^93^ FeMoco’s M-Fe5 bond polarizes net α-spin density toward the Mo-site *t*_2g_ (d_*xy*_) orbital, while the remaining M-Fe6 and -Fe7 spin-polarized bonds exhibit the opposite sign, with the d_*xz*_ and d_*yz*_ orbitals carrying net β-spin density. Figure 3 highlights the distinct spin-polarizations of FeMoco’s Mo–Fe bonds, reflecting our previous classification of a “non-Hund” (*S* = 1/2) Mo^3+^ site.^94,95^ Adopting a local *S* = 1/2 excited state provides the Mo-center with the proper spin-symmetry to form 2-center:2-electron bonds with each Fe-site.

FeFeco’s M- and adjacent Fe5-sites are ferromagnetically coupled, and similar to mixed-valency describing fractional oxidation states, the third electron of the M-Fe5 bond is delocalized between the two metal centers, populating a σ*-antibonding orbital that is fundamentally weaker than the covalent 2-center:2-electron bonds described. The unpaired electrons of FeFeco’s M-Fe6 and -Fe7 interactions are largely localized on their metal centers, indicative of weak-antiferromagnetic coupling. The covalency of the M-site is expected to further differentiate the Mo^3+^ and Fe^2+/3+^ oxidation states, as illustrated in their charge populations (Table S9). Cumulatively, FeFeco’s M-site is high-spin with a mixed-valence oxidation state of 2.5+. Holm has reported similar mixed-valency in Fe–S cubanes from ^57^Fe Mössbauer spectroscopy, featuring a distinct octahedral site.^96^ While FeFeco’s experimental ^57^Fe Mössbauer spectrum does not directly report M-site values, the δ (1.26 mm s^–1^), Δ*E*_Q_ (3.00 m s^–1^), and relative area (12.5%) assigned to an impurity are consistent with an octahedral, high-spin Fe^2+^ M-site.^37^ A summary of all metal sites’ local oxidation and spin states, along with their pairwise magnetic interactions relative to the BS-235M solution, is shown in Figure S4.

We characterized the properties of FeMoco’s and FeFeco’s secondary coordination spheres by the Extended Transition State-Natural Orbitals for Chemical Valence (ETS-NOCV) method, applying it to the Arg96 and Lys83 and Arg359 and Lys339 residues, which differ between the two cofactors. The ETS-NOCV method characterizes discrete noncovalent interactions between a specified residue and the cofactor. Figure 4 shows the orbital deformation densities for the analogous FeMoco and FeFeco cofactor-residue interactions. Regions of increased electron density (*purple*) and decreased electron density (*green*) illustrate polarization and charge transfer between the residue and the cofactor.

**Figure 4:**
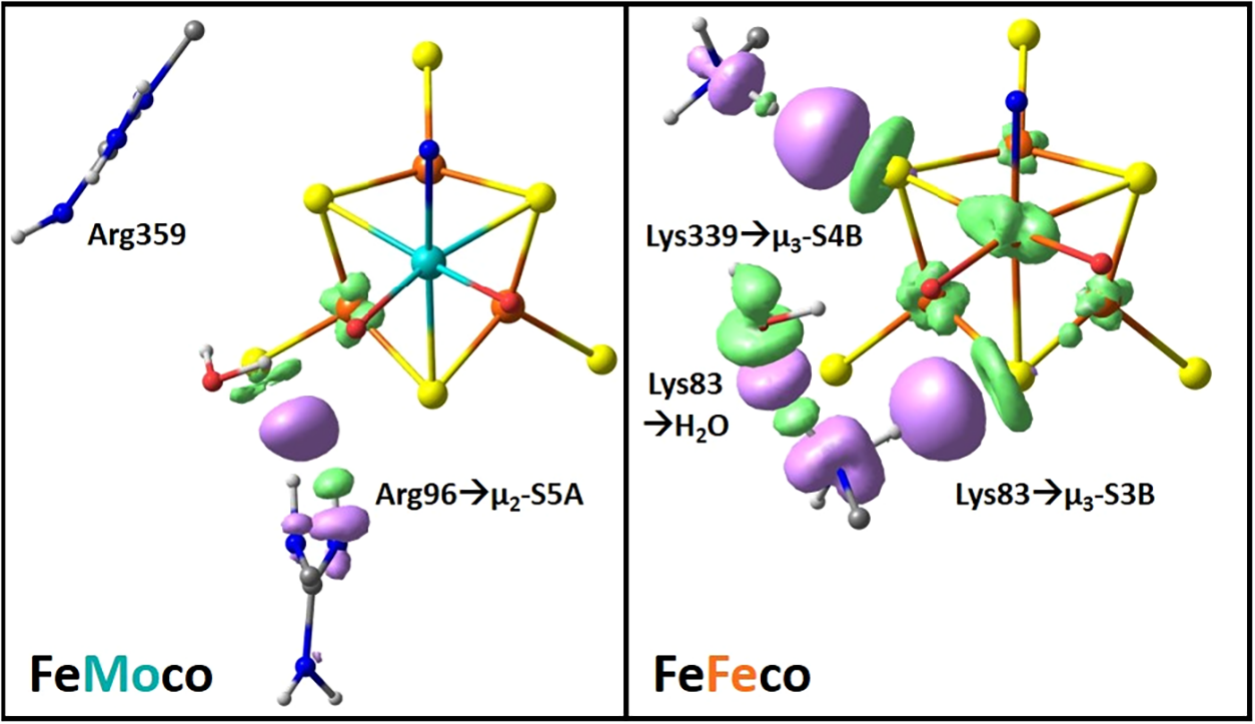
NOCV electron pair densities for the labeled residues with FeMoco (*left*) and FeFeco’s (*right*) E_0_ state. Their noncovalent interactions, resulting in an increase and decrease in electron density, are, respectively, purple and green. For clarity, only the [MFe_7_S_9_C] cluster and labeled residues with their acidic protons are included.

In FeMoco, Arg96 forms a hydrogen bond with the lone pair of μ_2_S5A, whereas Arg359 exhibits minimal orbital deformation, indicating that its interactions are primarily electrostatic. In contrast, FeFeco’s Lys83 and Lys359 direct their N–H bonds toward the heterocubane’s μ_3_S3B and -S4B sites, respectively, resulting in electron density accumulation between the acidic ammonium and basic sulfide groups, as shown in Figure 4. The deformation densities depict the charge transfer from sulfide→lysine *and* the proton transfer from lysine→sulfide.^97^ Coordinating both μ_3_S3B and -S4B, the lysine hydrogen bonds decrease electron density at FeFeco’s M and Fe7 centers. Lys83 also shows hydrogen bonding with a crystallographic water molecule, which should increase its acidity.

### E_1_ State Properties

We now turn to the possible structures of the E_1_ states of FeMoco and FeFeco. As discussed in the literature, the E_1_ state most likely features either a protonated bridging sulfide or an Fe-hydride, with their respective isomers shown in Figures S10 and S11. FeMoco residue substitutions support substrate binding and reduction on the face defined by the Fe2, Fe3, Fe6, and Fe7 centers, as highlighted in Figure 5.^98–100^ Consistent with previous reports, protonation of the μ_2_S2B site is the lowest-lying E_1_ state of FeMoco and FeFeco, with the isomers’ relative energies and assigned BS-solution provided in Tables S2–S3.^27,29^ Figure 5 highlights the difference in the μ_3_S3B E_1_ isomer, which lies +17.1 kcal mol^–1^ above the μ_2_S2B ground state in FeMoco but is only +3.5 kcal mol^–1^ higher in FeFeco. Using the TPSSh functional (Table S5), we find analogous trends in the E_1_ isomer energies, with μ_2_S2B as the most stable isomer for both cofactors and μ_3_S3B uniquely stabilized in FeFeco. Ryde’s recent QM/MM study does not assign FeFeco’s μ_3_S3B structure as a competitive E_1_ isomer, reporting it 14.8–18.2 kcal mol^–1^ above the μ_2_S2B ground state.^28^ We attribute the discrepancy to their choice of the BS-246 M solution, which we calculate to be +11.2 kcal mol^–1^ higher in energy than the BS-235M ground state (Table S4). Our differing findings highlight the inherent challenges of modeling nitrogenase catalytic states, which arise from the extensive permutations of their geometric and electronic configurations.

**Figure 5:**
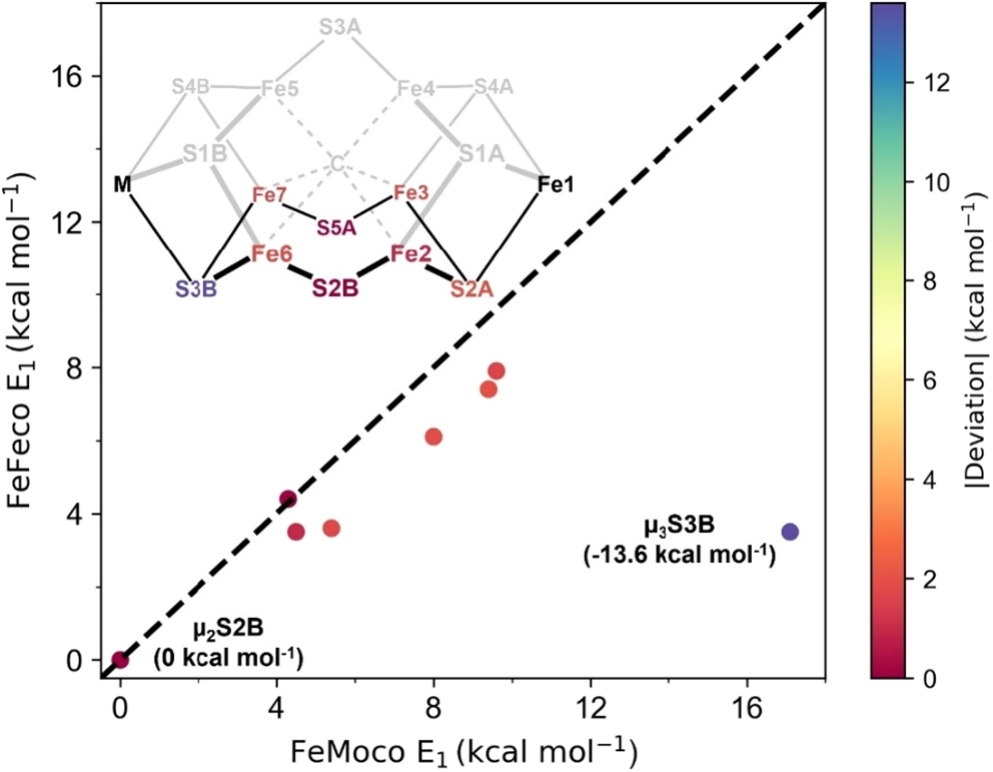
Relative QM/MM optimized energies of FeMoco (*x*-axis) and FeFeco’s (*y*-axis) E_1_ state isomers using the r^2^SCAN functional. The isomers are specific to the S–H and Fe–H structures in the cofactor’s 2/3/6/7 face (*inset*). The energies are relative to those of μ_2_S2B protonation. The color for the protonation site and data points corresponds to the absolute deviation between the cofactor’s analogous E_1_ isomers (color bar, *right*). The dashed black line, representing no deviation between cofactors, provides a guide for the eye. The stabilization of FeFeco’s μ_3_S3B isomer compared to FeMoco is highlighted.

As shown in Figure 6, protonation at the heterocubane core is accompanied by cleavage of the M-S3B bond. In both μ_3_S3B structures, the M-site adopts a square-pyramidal geometry with histidine bound axially. The μ_3_S3B localized orbitals assign both cofactors’ E_1_ states to a one-electron-reduced M-site, as shown in Figure S7. In FeMoco’s μ_3_S3B E_1_ isomer, the Mo^2+^ center preserves its covalent metal–metal bonds and local “non-Hund” configuration, which is inconsistent with our previous spectroscopy showing that the Mo^3+^ oxidation state is conserved in the E_1_ state.^26^ In FeFeco’s μ_3_S3B E_1_ isomer, the M-site is one-electron reduced, with a Fe^1+/2+^ mixed-valence oxidation state delocalized through ferromagnetic coupling to the Fe5 site. In both FeMoco and FeFeco, reduction of the μ_2_S2B site is assigned to the heterocubane’s Fe6-site (Figure S8).

**Figure 6:**
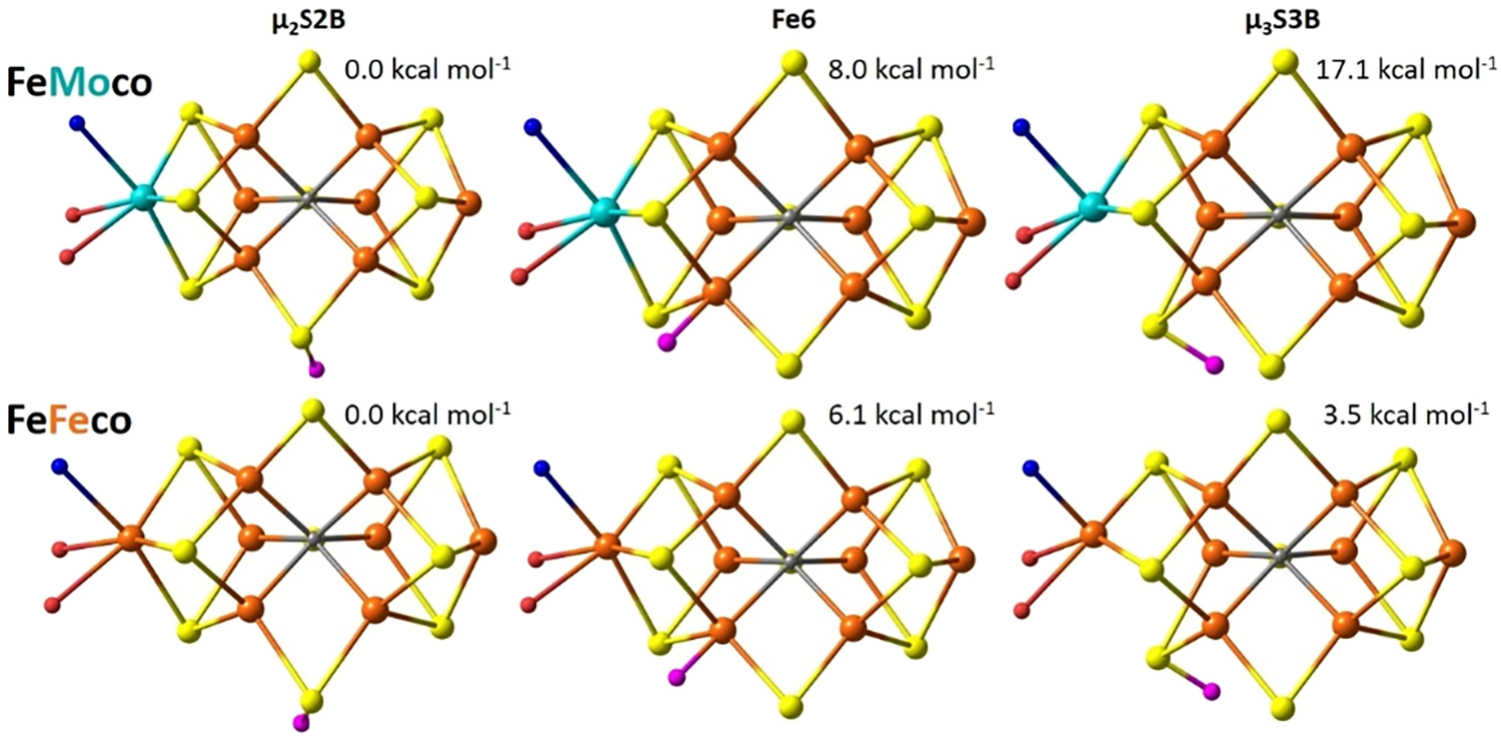
QM/MM optimized structures and relative energies for the FeMoco (*top*) and FeFeco’s (*bottom*) μ_2_S3B (*left*), Fe6 (*center*), and μ_3_S3B (*right*) E_1_ isomers. The added proton of the E_1_ state is shown as pink.

The E_1_ model proposed by Hoffman and co-workers invokes Fe-hydrides. The calculated Fe-hydride E_1_ structures exhibit local trigonal bipyramidal geometries, with the terminal hydride coordinated *trans*- to the carbide. Although Hoffman and co-workers proposed a μ_2_-hydride, we do not find such a structure to be stable for either FeFeco or FeMoco.^30^ In both FeMoco and FeFeco terminal hydrides are preferentially stabilized at the Fe2/Fe3 sites rather than the Fe6/Fe7 sites (∼3.5 kcal mol^–1^) within their respective homo- and heterocubanes, with the most stable hydride having an energy comparable to the μ_3_S3B isomer. Our orbital analysis suggests the Fe-centers are insufficiently reduced to readily facilitate hydride formation in the cofactor’s E_1_ state (Figure S9). We observe minimal variation (∼1.5 kcal mol^–1^) in the Fe-hydride energies between the two cofactors. We note that the calculated terminal hydride models should produce axial symmetry in the hyperfine coupling tensor. Such axial symmetry was not observed in the ^1,2^H ENDOR characterization of FeMoco’s E_4_ state^35^ (the most studied reduced FeMoco intermediate), and ^1,2^H ENDOR experiments have not been reported for FeFeco.

The low-lying μ_3_S3B isomer in FeFeco therefore provides an alternative explanation for the photoisomerization observed in the photolysis EPR experiments. The distinct reaction coordinates connecting FeMoco and FeFeco’s μ_3_S3B E_1_ states to the more stable μ_2_S2B isomer were calculated using the Nudged Elastic Band-Climbing Image (NEB-CI) method. The corresponding free energies are shown in Figure 7, and the full transition state structures and imaginary frequencies are presented in Figure S15. We assign the μ_3_S3B isomer as an initial protonation site on the cofactor, due to the proximal water molecules^41^ and acidic residues, in contrast to the μ_2_S2B site, which is located in a more hydrophobic region. The free energies (Δ*G*) are relative to the cofactor’s lowest-lying μ_2_S2B isomer.

**Figure 7:**
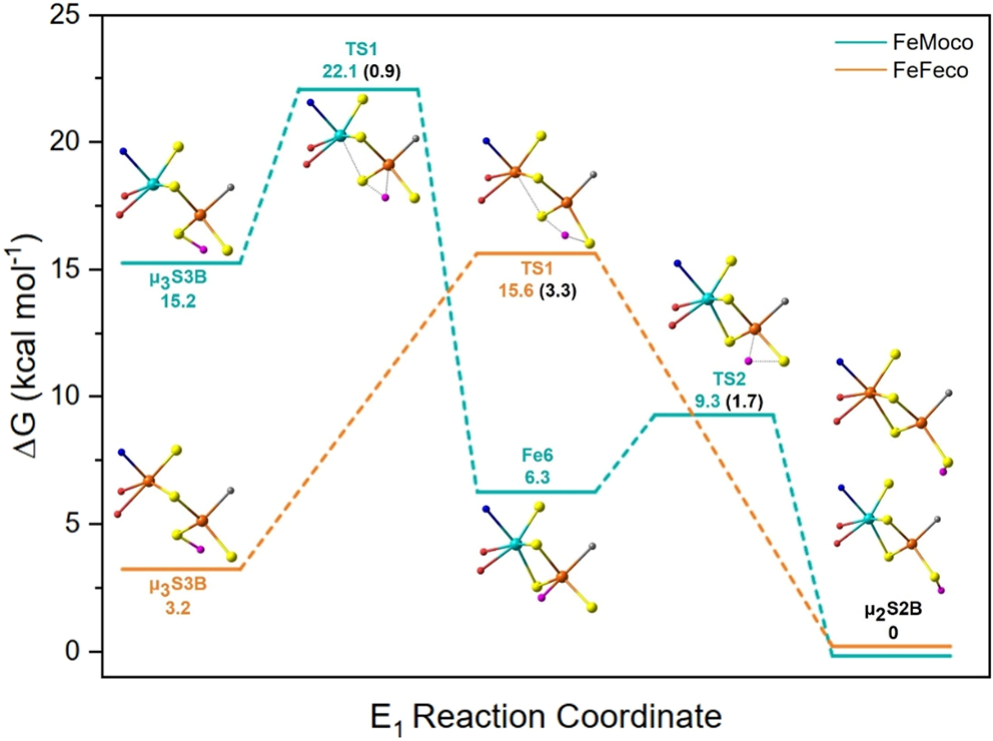
Relative energies of FeMoco (*blue*) and FeFeco’s (*orange*) transition states and intermediates that connect their μ_3_S3B and μ_2_S2B E_1_ states. All energies are referenced to the cofactors’ μ_2_S2B isomer assigned in Figure 6. Only the cofactors’ M- and Fe6-primary coordination spheres are highlighted for clarity, and the added proton of the E_1_ state is shown as pink. Bond formation and dissociation are illustrated as a dashed line. Both FeMoco’s TS1 and TS2 structures have a BS-346M state, and FeFeco’s TS1 state has a BS-2467 solution. The transition state’s room temperature KIEs are provided in black in parentheses.

FeMoco exhibits a two-step process with the Fe6 isomer connecting the sulfide-protonated states. The Fe-hydride is thermodynamically favored due to the large instability of the μ_3_S3B isomer. The corresponding transition state, TS1, (Δ*G* ^‡^ = +6.9 kcal mol^–1^), involves twisting the S3B–H bond toward the Fe6 center. A smaller reaction barrier (Δ*G* ^‡^ = +3.0 kcal mol^–1^) separates Fe6 and the preferential μ_2_S2B isomer. These results suggest that FeMoco’s μ_2_S2B isomer is thermodynamically isolated from the initial μ_3_S3B protonated state. The calculated KIEs of FeMoco’s TS1 and TS2 states are small, with values of 0.9 and 1.7, respectively. Uniquely, a single transition state connects FeFeco’s μ_3_S3B and μ_2_S2B isomers, involving a concerted S–H proton transfer without formation of an Fe-hydride (Tables S16–S18), consistent with Hoffman’s photolysis data, which similarly supports a single kinetic state connecting the E_1_ isomers without an intermediate. FeFeco’s reaction barrier (Δ*G* ^‡^ = +12.4 kcal mol^–1^) corresponds to a KIE of 3.3, which is consistent with the experimental range of 2.0–2.8. For comparison, FeMoco’s E_2_ state H_2_ production has an experimental KIE of 2.7, calculated as 3.2 in other studies.^36,101^ More importantly than the absolute values, our calculations illustrate that transitions involving only the S–H states can exhibit pronounced KIEs. The calculated saddle point shows the E_1_ state’s H-center forming a nearly symmetric bridge between the S3B (1.70 Å) and S2B (1.78 Å) sites. Localized orbital analysis indicates that the transition state is best described as a proton transfer between the sulfur lone pairs. However, our study does not discern whether FeFeco can have an analogous reaction coordinate to FeMoco, where the higher lying Fe-hydride is an intermediate connecting the μ_3_S3B and μ_2_S2B isomers. The transition state is accompanied by contraction of the M-S3B distance, restoring the octahedral geometry at the homocitrate-bound iron (M).

## Conclusions

Our study underscores the need for caution when directly comparing distinct nitrogenase systems. Previously unaddressed in their resting state characterizations, we detail the electronic structure origins underlying the structural deviations of the M-site in FeMoco and FeFeco, considering both their primary and secondary coordination spheres. Far from equivalent, we find that the reaction coordinates connecting the cofactors’ E_1_ isomers are disparate. The stability of a 5-coordinate, reduced M-site, which is unique to FeFeco’s μ_3_S3B isomer, favors a concerted pathway toward the previously detailed belt-sulfide-protonated μ_2_S2B structure. The comparative instability of FeMoco’s analogous μ_3_S3B isomer may be consistent with the sluggish proton-transfer kinetics reported for model Mo–Fe–S clusters described above.^49^ Ultimately, we show that thermal and photolability in nitrogenase cofactors are not mutually exclusive with sulfur-protonated intermediates. Such arguments are foundational to the mechanistic assignment of nitrogenase, in which Fe-bridging hydrides facilitate nitrogen fixation.^32^ While both FeMoco and FeFeco share a lowest-lying E_1_ state involving protonation of a belt-sulfide adjacent to the carbide core, FeFeco’s unique opening of the heterocubane framework exposes a coordinatively unsaturated, reduced Fe-site that is potentially competent for the enzyme’s enhanced H^+^ reduction and its ability to reduce CO_2_ to CH_4_.

## Supplementary Material





## Data Availability

All relevant data generated and analyzed during the study will be made available in the Edmond Open Research Data Repository 10.17617/3.KTDXGN.

## Notes

Additional geometric structural data, determination of broken-symmetry solutions, population analysis, localized orbital analysis, E_1_ isomer and conformational analysis and transition state analysis. The QM-region coordinates for select optimized E_0_ and E_1_ states for FeMoco and FeFeco are provided. The NEB-CI obtained reaction coordinates for FeMoco and FeFeco’s E_1_ transition states are provided as animations (PDF)
xyz (ZIP)

## Abbreviations

QM/MM: Quantum Mechanics/Molecular Mechanics
DFT: Density Functional Theory
BS: Broken Symmetry
