# Supplementary material for: Unravelling Catalytic Divergence in Mo- and Fe-Only Nitrogenases: The Role of the Heterometal-Site and Protein Environment from QM/MM Insights
Source: J Am Chem Soc. 2025 Dec 16;147(52):48416–26. doi: 10.1021/jacs.5c20796 (PMC12766731; doi:10.1021/jacs.5c20796)
Supplement: Supplementary file 1 [file ja5c20796_si_001.pdf]

SUPPORTING INFORMATION

for

Unravelling Catalytic Divergence in Mo- and Fe-only Nitrogenases: The Role of the Heterometal-site and Protein Environment from QM/MM Insights

Justin P. Joyce [a], Ragnar Bjornsson [b]* and Serena DeBeer [a]*

[a] *Max Planck Institute for Chemical Energy Conversion, 45470 Mülheim an der Ruhr, Germany*

[b] *Univ. Grenoble Alpes, CNRS, CEA, LCBM (UMR 5249), F-38000 Grenoble, France*

*Email: ragnar.bjornsson@cea.fr

*Email: serena.debeer@cec.mpg.de

Table of Contents:

I. Computational Setup (1)
II. Description of Broken Symmetry States (2)
III. Geometric Structure Analysis (6)
IV. Hirshfeld Spin Population Analysis (7)
V. Localized Orbital Analysis (8)
VI. Conformational Description of E_1 Isomers (11)
VII. Description of Transition States (14)
VIII. Total Electronic Energies (16)
IX. References (17)

SI. Computation Setup

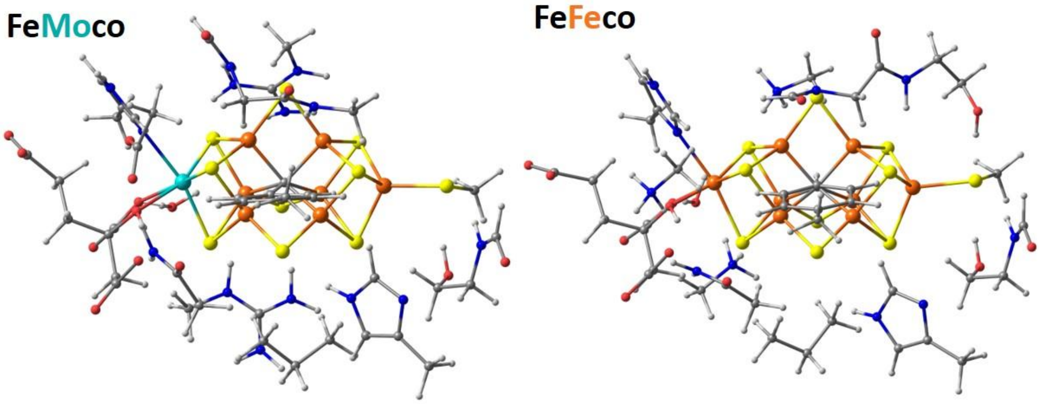

Figure S1. The QM-region of FeMoco (*left*) and FeFeco (*right*), illustrated in their BS-235M optimized E_0 state. The residue identities for FeMoco include Val70, Arg96, Gln191, His195, Cys275, Ser278, Gly356, Gly357, Arg359, Glu380, Phe381, His442 and HOH519 and totals 168 atoms, when including the link atoms. The residue identities of FeFeco are Val57, Lys83, Gln176, His180, Cys257, Ser260, Gly337, Ser338, Lys339, Lys361, Phe362, His423 and HOH887 and totals 163 atoms, when including the link atoms.

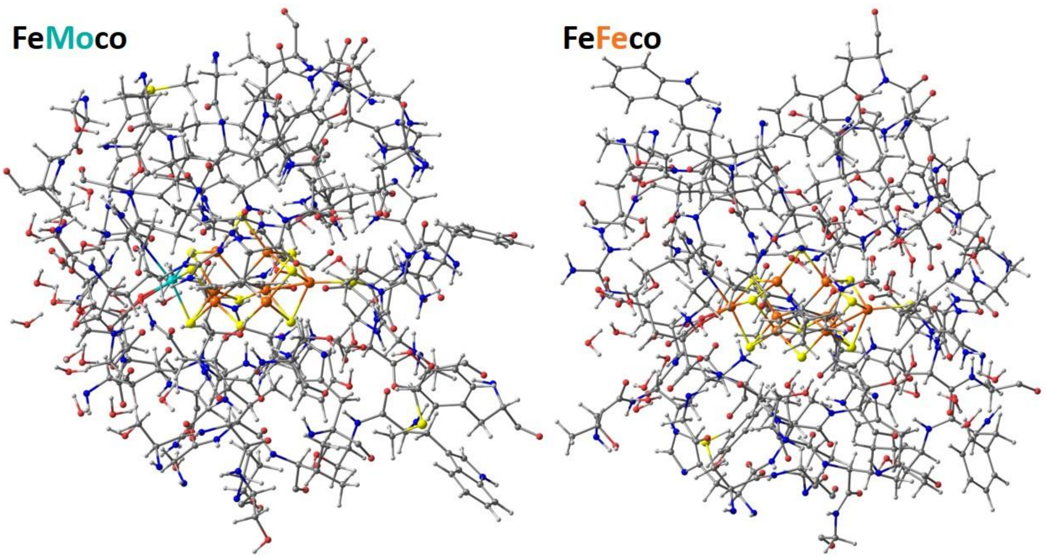

Figure S2. The respective 1002 and 959 atom active region of FeMoco (*left*) and FeFeco (*right*), illustrated in their BS-235M optimized E_0 state.

<u>SII. Description of Broken Symmetry States:</u>

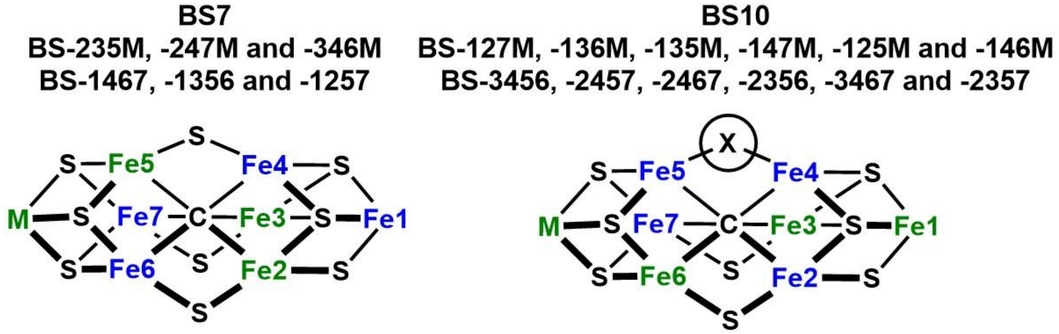

Figure S3. The spin topology of the BS7 (*left*) and -10 (*right*) class of broken-symmetry solutions. The M-site's homocitrate ligand and coordinated histidine are omitted to highlight the cofactor's C_3-symmetry. The metal-sites of dominant α-(up) and β-(down) spin character are respectively assigned blue and green. The BS-labels for this manuscript's notation are provided. The perturbation of a μ_2-sulfide is highlighted as "X" for the BS10 solution.

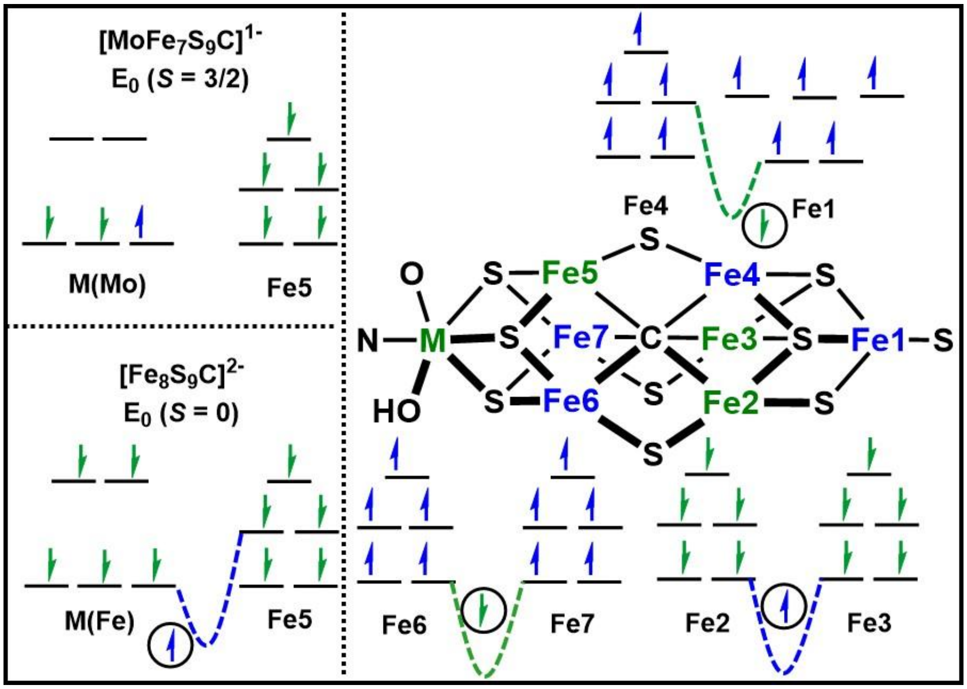

Figure S4. The BS-235M QM/MM assigned oxidation and spin states and pairwise magnetic interactions for the metal-sites in FeMoco and FeFeco's E_0 state. Metal-sites of dominant α-(up) and β-(down) spin character are respectively assigned blue and green. The mixed-valence (ferromagnetic) interactions between Fe-sites is illustrated with a dashed line. The cofactor's M-site and bonding interaction to Fe5 (*left*) are differentiated.

Table S1. Relative QM/MM energies of the geometry optimized E_0 Broken Symmetry solutions of FeMoco and FeFeco

BS-Solution	FeMoco $\Delta E_{QM/MM}$ (kcal mol^{-1})	FeFeco $\Delta E_{QM/MM}$ (kcal mol^{-1})
BS-235M	0	0
BS-247M	1.49	1.34
BS-346M	0.13	1.09

Table S2. The relative energies of select BS-solutions for FeMoco (M_s = 2) E_1 isomers in the cofactors 2/3/6/7 plane with respect to the μ_2S2B (BS-346M) structure.

E_1 Configuration (FeMoco)	Lowest Energy BS-Solution	BS-235M $\Delta E_{QM/MM}$ (kcal mol^{-1})	BS-247M $\Delta E_{QM/MM}$ (kcal mol^{-1})	BS-346M $\Delta E_{QM/MM}$ (kcal mol^{-1})
μ_2S2B	BS-346M	1.75	3.44	0
μ_3S3B	BS-235M	17.14	18.68	19.86
Fe6	BS-346M	10.66	12.22	7.99
Fe7	BS-247M	10.74	9.57	11.97
μ_2S5A	BS-247M	6.17	4.27	6.15
Fe2	BS-235M	4.53	6.44	13.99
Fe3	BS-235M	5.40	14.86	5.92
μ_3S2A	BS-235M	9.54	14.57	14.89

Table S3. The relative energies of select BS-solutions for FeFeco's E_1 isomers (M_s = 1/2) in the 2/3/6/7 plane with respect to the μ_2S2B (BS-2467) structure.

E_1 Configuration (FeFeco)	Lowest Energy BS-Solution	BS-235M $\Delta E_{QM/MM}$ (kcal mol^{-1})	BS-247M $\Delta E_{QM/MM}$ (kcal mol^{-1})	BS-346M $\Delta E_{QM/MM}$ (kcal mol^{-1})
μ_2S2B	BS-2467	2.07	2.24	1.33
μ_3S3B	BS-235M	3.51	6.10	6.98
Fe6	BS-346M	12.36	11.93	6.06
Fe7	BS-247M	14.56	7.85	14.67
μ_2S5A	BS-3467	6.60	5.43	6.46
Fe2	BS-235M	3.48	4.90	15.84
Fe3	BS-235M	3.59	15.67	5.25
μ_3S2A	BS-235M	7.39	10.20	12.97

Table S4. The relative energies of the complete seventy BS-solutions for FeFeco's μ_3S3B E_1 isomer (S = 1/2). The relative energies are with respect to the structure's lowest energy BS-solution (BS-235M).

BS-solution	$\Delta E_{QM/MM}$ (kcal mol^{-1})
BS-567M	51.27
BS-1234	49.17
BS-234M	12.75
BS-1567	8.30
BS-123M	16.19
BS-4567	38.80
BS-124M	13.79
BS-3567	9.31
BS-134M	12.84
BS-2567	8.54
BS-257M	10.79
BS-1346	12.22
BS-356M	15.17
BS-1247	14.80
BS-467M	40.55
BS-1235	12.65
BS-256M	14.60
BS-1347	8.47
BS-357M	14.37
BS-1246	17.21
BS-367M	11.41
BS-1245	8.30
BS-456M	17.00
BS-1237	16.90
BS-457M	17.63
BS-1236	9.03
BS-267M	46.06
BS-1345	49.09
BS-156M	11.44
BS-2347	9.78
BS-157M	11.41
BS-2346	8.82
BS-167M	10.28
BS-2345	8.04

BS-235M	0.00
BS-1467	5.78
BS-247M	2.59
BS-1356	9.00
BS-346M	3.47
BS-1257	11.07
BS-245M	8.15
BS-1367	8.12
BS-345M	9.43
BS-1267	11.17
BS-236M	11.21
BS-1457	10.07
BS-246M	11.16
BS-1357	10.90
BS-237M	10.10
BS-1456	10.51
BS-347M	11.20
BS-1256	12.35
BS-126M	14.21
BS-3457	11.04
BS-137M	13.40
BS-2456	11.35
BS-145M	13.49
BS-2367	8.57
BS-127M	10.83
BS-3456	10.57
BS-136M	11.30
BS-2457	9.39
BS-135M	7.39
BS-2467	6.62
BS-147M	8.46
BS-2356	8.35
BS-125M	8.05
BS-3467	5.74
BS-146M	8.58
BS-2357	8.11

Table S5. The FeMoco and FeFeco E_1 optimized isomers in the 2/3/6/7 face. The energies (TPSSh/MM) correspond to the optimized structures of their lowest-lying BS-solution of their respective M_s = 2 and 1/2 states.

E_1 isomer	FeMoco (kcal mol^{-1})	FeFeco (kcal mol^{-1})
μ_2S2B	0.00	0.00
μ_2S5A	4.63	4.12
μ_3S3B	21.87	1.92
μ_3S2A	8.56	6.14
Fe6	10.12	7.35
Fe7	12.24	9.49
Fe2	7.07	4.19
Fe3	7.59	3.86

SIII. Geometric Structure Analysis

Table S6. Metal-Metal Distances of the experimental crystallographic structure of FeMoco (PDB 3U7Q)[1] and FeFeco's (PDB 8BOQ)[2] E_0 state. Their difference is provided, a positive value assigning a longer distance in FeFeco.

Metal-Metal Distance	FeMoco (Å) 3U7Q	FeFeco (Å) 8BOQ	$\Delta_{FeFeco,FeMoco}$ (Å)
M-Fe5	2.73	2.80	+0.07
M-Fe6	2.60	2.93	+0.33
M-Fe7	2.68	2.84	+0.16
Fe1-Fe2	2.67	2.68	+0.01
Fe1-Fe3	2.67	2.61	-0.06
Fe1-Fe4	2.66	2.65	-0.01
Fe2-Fe3	2.67	2.62	-0.05
Fe2-Fe4	2.65	2.65	0.00
Fe2-Fe6	2.58	2.61	+0.03
Fe3-Fe4	2.64	2.65	+0.01
Fe3-Fe7	2.59	2.63	+0.04
Fe4-Fe5	2.61	2.60	-0.01
Fe5-Fe6	2.63	2.62	-0.01
Fe5-Fe7	2.63	2.66	+0.03
Fe6-Fe7	2.60	2.61	+0.01

Table S7. Metal-Metal Distances of the QM/MM geometry optimized BS-235M solutions of FeMoco (M_s = 3/2) and FeFeco (M_s = 0) E_0 state. Their difference is provided, a positive value assigning a longer distance in FeFeco.

Metal-Metal Distance	FeMoco (Å) BS-235M	FeFeco (Å) BS-235M	$\Delta_{FeFeco,FeMoco}$ (Å)
M-Fe5	2.72	2.79	+0.07
M-Fe6	2.64	2.93	+0.29
M-Fe7	2.63	2.85	+0.22
Fe1-Fe2	2.65	2.68	+0.03
Fe1-Fe3	2.63	2.60	-0.03
Fe1-Fe4	2.64	2.65	+0.01
Fe2-Fe3	2.65	2.62	-0.03
Fe2-Fe4	2.64	2.65	+0.01
Fe2-Fe6	2.58	2.61	+0.03
Fe3-Fe4	2.62	2.66	+0.04
Fe3-Fe7	2.59	2.59	0.00
Fe4-Fe5	2.58	2.60	+0.02
Fe5-Fe6	2.62	2.65	+0.03
Fe5-Fe7	2.64	2.64	0.00
Fe6-Fe7	2.55	2.61	+0.06

Table S8. Cubane Volume Elements of the QM/MM geometry optimized BS-235M solutions of FeMoco (M_s = 3/2) and FeFeco (M_s = 0) E_0 state. Their difference is provided, a positive value assigning a larger volume in FeFeco.

Tetrahedron Volume[3] (Cubane Sites)	FeMoco ($Å^3$) BS-235M	FeFeco ($Å^3$) BS-235M	$\Delta_{FeFeco,FeMoco}$ ($Å^3$)
MFe_3 (M, Fe5, Fe6, Fe7)	2.149	2.419	+0.270 (12.6%)
Fe_4 (Fe1, Fe2, Fe3, Fe4)	2.163	2.176	+0.013 (0.6%)
$S(B)_3C$ (S1B, S3B, S4B, C)	4.822	4.960	+0.043 (0.9%)
$S(A)_3C$ (S1A, S2A, S4A, C)	4.887	4.930	+0.138 (2.9%)

SIV. Hirshfeld Population Analysis

Table S9. The Hirshfeld charge population of the metal center's in the QM/MM optimized geometries of FeMoco and FeFeco's E_0, μ_2S2B and μ_2S2B E_1 isomers. A positive and negative value respectively assign cationic and anionic charge.

Site	FeMoco E_0 BS-235M	FeFeco E_0 BS-235M	FeMoco μ_2S2B BS-346M	FeFeco μ_2S2B BS-2467	FeMoco μ_3S3B BS-235M	FeFeco μ_3S3B BS-235M
M	0.50	0.19	0.49	0.18	0.47	0.10
Fe1	0.08	0.07	0.09	0.06	0.08	0.07
Fe2	0.10	0.11	0.09	0.06	0.10	0.10
Fe3	0.09	0.08	0.09	0.08	0.08	0.07
Fe4	0.07	0.09	0.06	0.09	0.07	0.09
Fe5	0.08	0.10	0.04	0.07	0.08	0.07
Fe6	0.04	0.11	0.00	0.10	0.02	0.10
Fe7	0.03	0.08	0.03	0.10	0.01	0.08

Table S10. The Hirshfeld spin population of the metal center's in the QM/MM optimized geometries of FeMoco and FeFeco's E_0, μ_2S2B and μ_2S2B E_1 isomers. A positive and negative values respectively assign α- and β-spin.

Site	FeMoco E_0 BS-235M	FeFeco E_0 BS-235M	FeMoco μ_2S2B BS-346M	FeFeco μ_2S2B BS-2467	FeMoco μ_3S3B BS-235M	FeFeco μ_3S3B BS-235M
M	-0.32	-3.51	-0.25	+3.49	+0.54	-3.06
Fe1	+3.16	+3.13	+3.17	+3.13	+3.16	+3.14
Fe2	-2.78	-2.87	+2.94	-2.85	-2.81	-2.88
Fe3	-2.83	-2.85	-2.84	3.04	-2.85	-2.85
Fe4	+3.01	+3.06	-2.86	-2.92	+3.02	+3.09
Fe5	-2.76	-3.04	+2.67	+2.97	-2.77	-2.91
Fe6	+2.56	+2.98	-2.00	-3.05	+2.63	+3.02
Fe7	+2.57	+2.95	+2.61	-3.04	+2.56	+2.99

SV. Localized Orbital Analysis

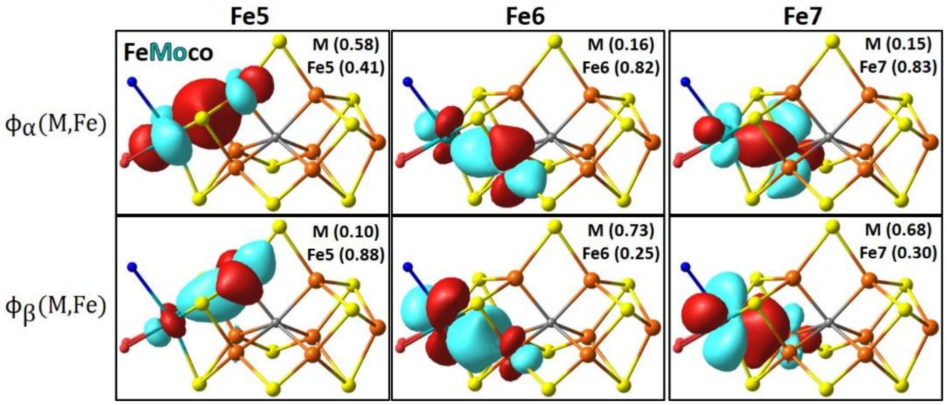

Figure S5. The PM-localized orbitals of FeMoco's E_0 state (BS-235M) descriptive of the M-Fe interactions. The one-electron orbitals of α(up) and β(down) spin are respectively shown at the *top* and *bottom*. Mulliken electron density population is included for the M- and Fe-site.

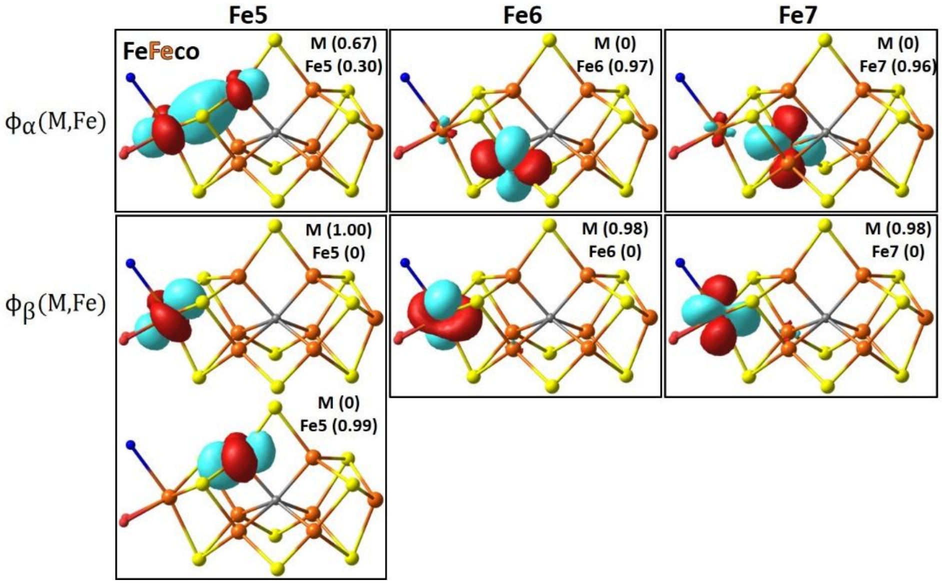

Figure S6. The PM-localized orbitals of FeFeco's E_0 state (BS-235M) descriptive of the M-Fe interactions. The one-electron orbitals of α(up) and β(down) spin are respectively shown at the *top* and *bottom*. The Mulliken electron density population is included for the M- and Fe-site.

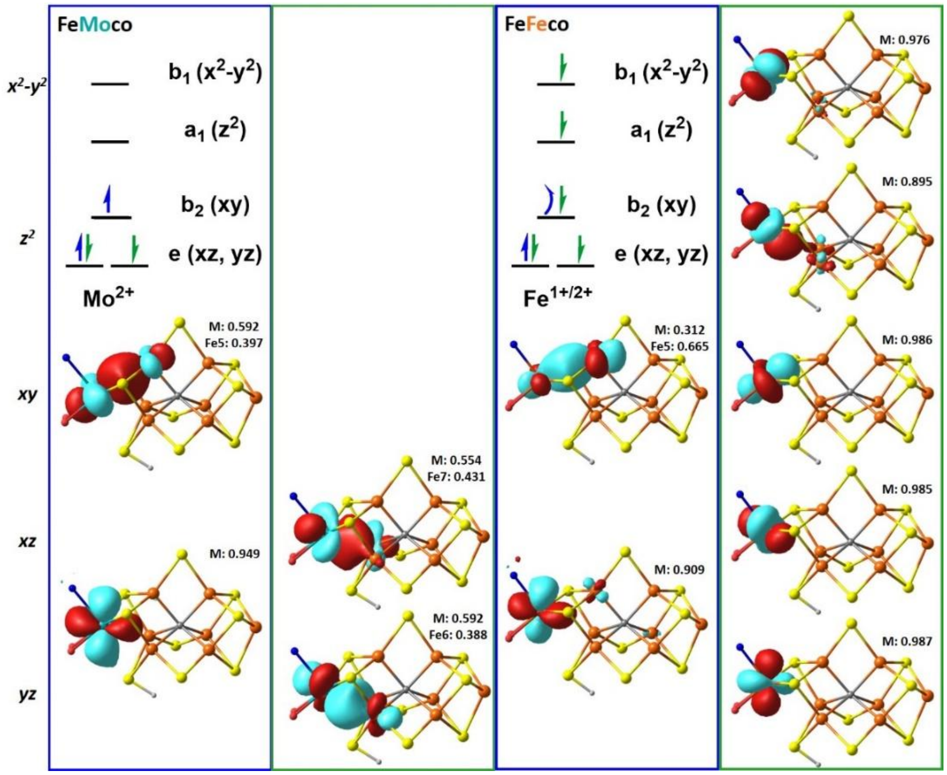

4

Figure S7. The PM-localized orbitals at FeFeco's M-site in its QM/MM optimized (BS-235M) μ_3S3B isomer. The orbitals in the blue and green rectangles are respectively α- and β-spin. The Mulliken population is provided. The qualitative orbital diagrams are labeled with respect to an ideal C_{4v} symmetry in a square pyramidal complex. The curved arrow assigns the delocalized electron of the mixed-valence system.

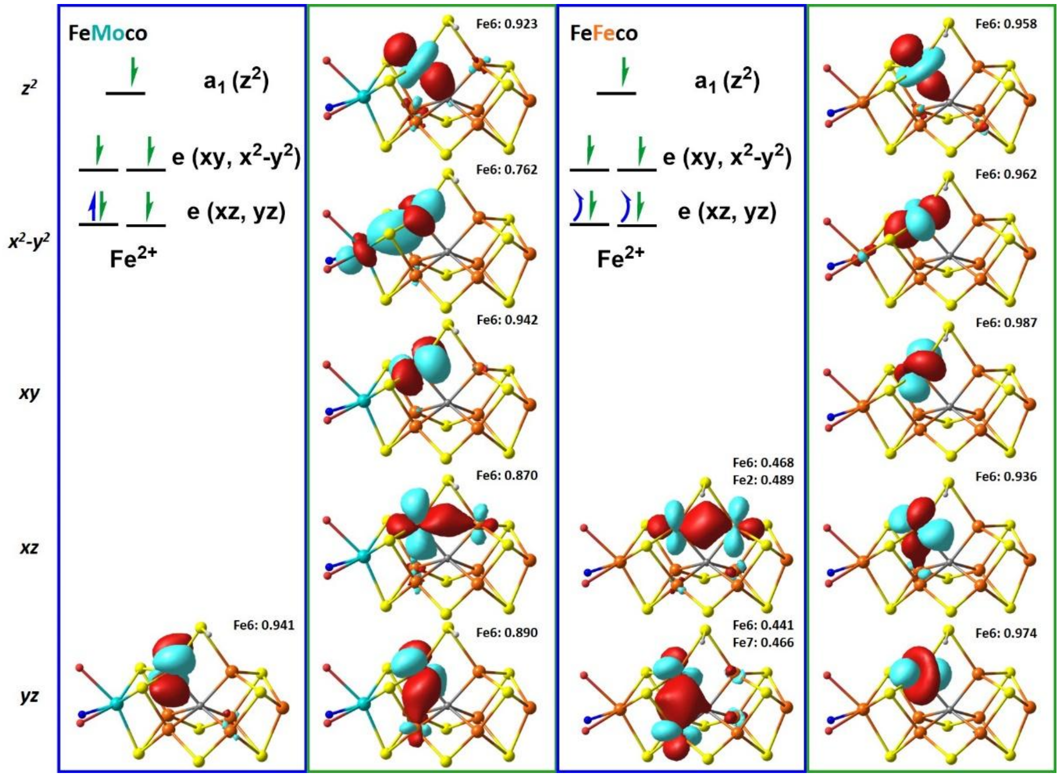

Figure S8. The PM-localized orbitals at FeMoco's Fe6-site in its QM/MM optimized (BS-346M) μ_2S2B isomer. The orbitals in the blue and green rectangles are respectively α- and β-spin. The Mulliken population is provided. The qualitative orbital diagrams are labeled with respect to an ideal C_{3v} symmetry in a trigonal pyramidal complex. The curved arrow assigns the delocalized electron of the mixed-valence system.

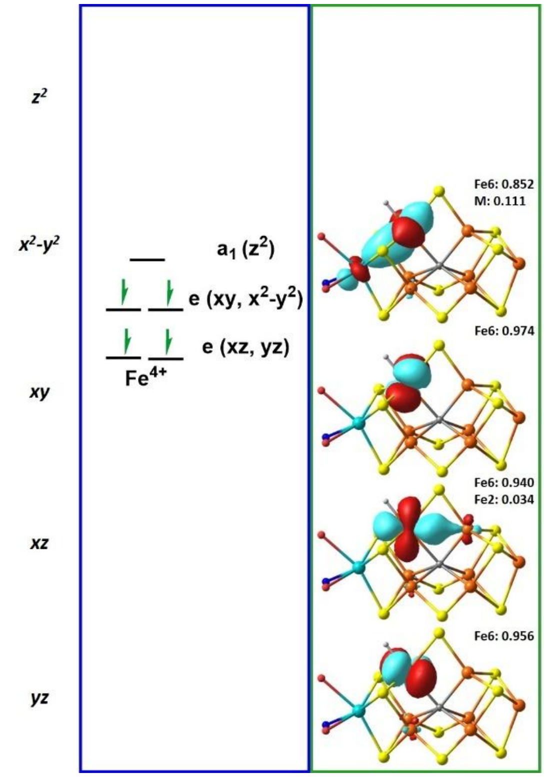

Figure S9. The PM-localized orbitals at FeMoco's Fe6-site in its QM/MM optimized (BS-346M) Fe6 isomer. The orbitals in the blue and green rectangle are respectively α- and β-spin. The Mulliken population is provided. The qualitative orbital diagram (*upper left*) are labeled with respect to an ideal C_{3v} symmetry.

SVI. Geometric- and Stereo-isomers of E_1 States

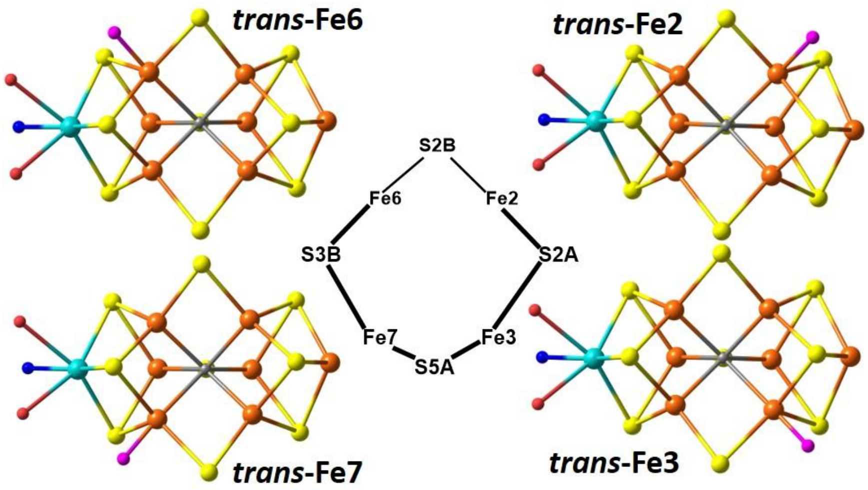

Figure S10. (*center*) The atom centers included in the 2/3/6/7 face for possible E_1 structures. (*outside*) The QM/MM optimized geometries of the Fe-hydride containing (pink) E_1 state in FeMoco. Analogous structures are found in FeFeco's E_1 states

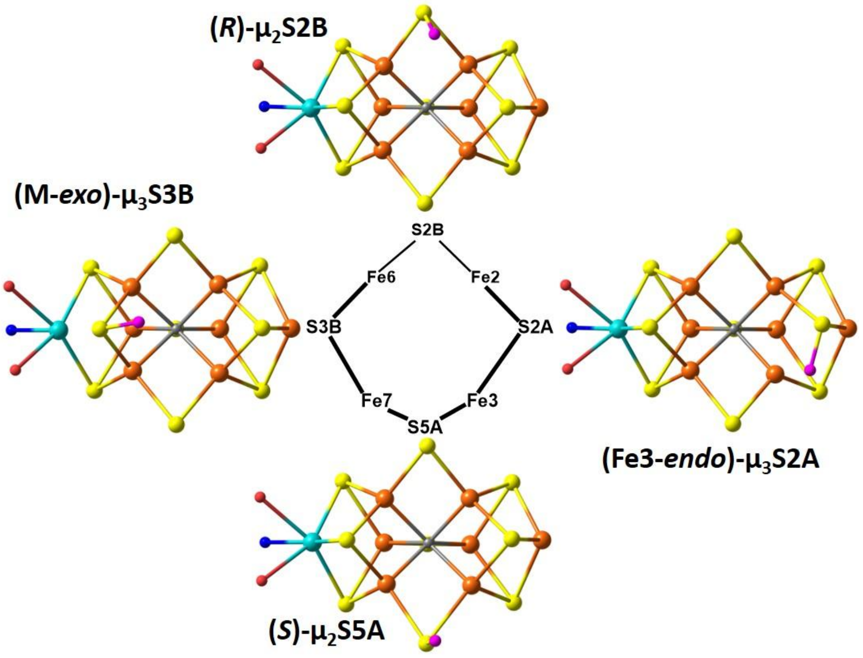

Figure S11. (*center*) The atom centers included in the 2/3/6/7 face for possible E_1 structures. (*outside*) The QM/MM optimized geometries of the sulfur-protonated (pink) E_1 isomers in FeMoco. The stereochemistry of the protonated sulfur-site is provided. Analogous isomers are found in FeFeco's E_1 states.

μ_2-sulfide Protonation Conformations.

The μ_2-sulfide$^-$ sites have two possible stereochemistry with their protonation. Their conformations are differentiated with *R*- and *S*-nomenclature in Figure S12, assigning the hetero-cubane site priority. Total, there are six μ_2S configurations in the cofactor. The (*R*)-μ_2S2B and (*S*)-μ_2S5A configurations are in the cofactor's 2/3/6/7 face, shown in Figure S11. The (*R*)-μ_2S2B conformation is referenced as S2B(5) in Ryde's earlier study, the S2B-H bond being directed towards the μ_2S5A site.[4,5] We note that the (*S*)-μ_2S2B conformation is calculated as 2.1 kcal mol^{-1} more stable than (*R*)-μ_2S2B detailed in the manuscript. Ryde also reported a relative stability of 1.7-2.2 kcal mol^{-1} FeFeco's analogous S2B(3) and S2B(5) conformations.[5]

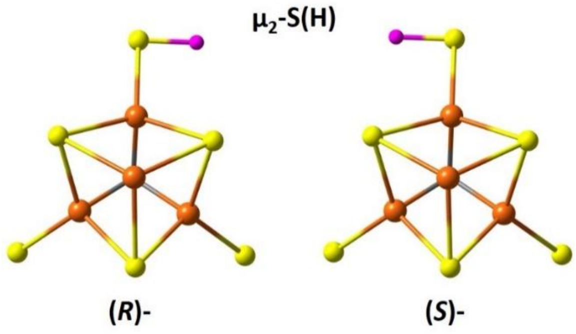

Figure S12. The possible stereochemistry for μ_2-sulfide protonation, the (*R*)- and (*S*)- conformations oriented along the cofactor's approximate C_3-axis.

Fe-hydride Conformations.

The Fe-centers in the $\{Fe_6C\}$ core have three hydride conformations (Figure S13). The E_1 configurations can be *trans*- or *cis*-geometric isomers with respect to the interstitial carbide. The *cis*-conformations can be (*R*)- or (*S*)-

stereoisomers, assigning priority to the hetero-cubane. The *cis*-conformations are assigned as μ_2-hydrides if their distance to adjacent Fe-sites deviates less than 0.2 Å. We report that the *trans*-conformations are energetically preferred to the *cis*-geometries of the Fe2, Fe3, Fe6 and Fe7 sites considered in the manuscript, shown in Figure S10. Our calculated relative energy of 3.5 kcal mol^{-1} for FeFeco's Fe2 E_1 isomer is consistent with the 4.1-4.5 kcal mol^{-1} value reported by Ryde.[5]

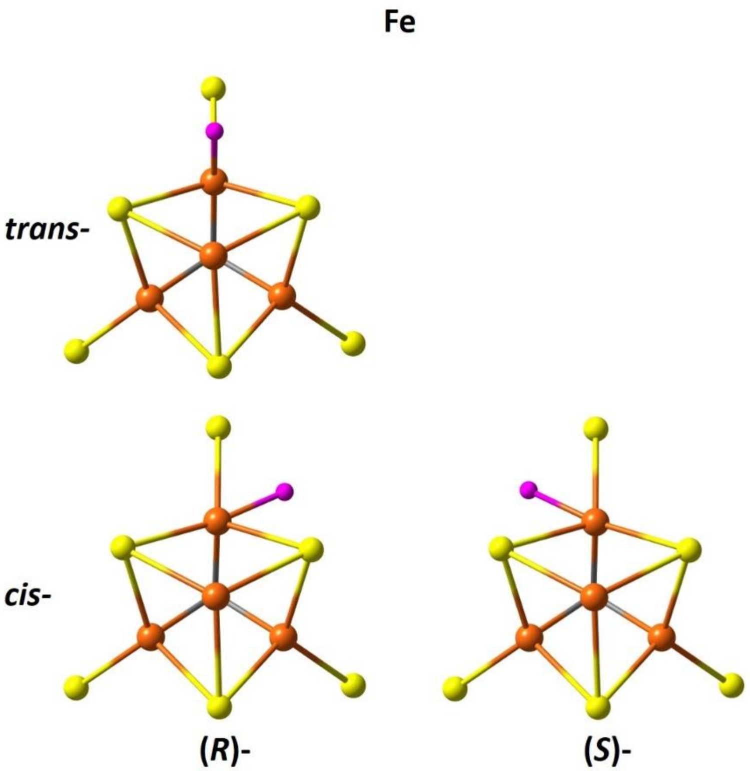

Figure S13. The possible stereochemistry for Fe-hydride formation, shown along the cofactor's approximate C_3-axis.

μ_3-sulfide Protonation Conformations.

The cubane's μ_3-sulfide sites have six protonated conformations, shown in Figure S14. The protonation incites dissociation of a sulfur-metal bond. The S-H bond has *endo*- or *exo*-stereochemistry with respect to its orientation towards or away from the dissociated metal-center.[6] There are 36 μ_3S configurations in the nitrogenase cofactor. There are six possible μ_3S E_1 configurations in the cofactor's 2/3/6/7 face: μ_3S2A(Fe2-*endo*), -S2A(Fe3-*endo*), -S2A(Fe1-*exo*), -S3B(Fe6-*endo*), -S3B(Fe7-*endo*) and -S3B(M-*exo*). We consider μ_3S conformations whose structures are stable following optimization of their BS-235M, -247M and -346M solutions. Both FeMoco and FeFeco's S3B(Fe6-*endo*) conformation relaxes to the hydride-containing Fe6 structure when optimized with the BS-346M solution.

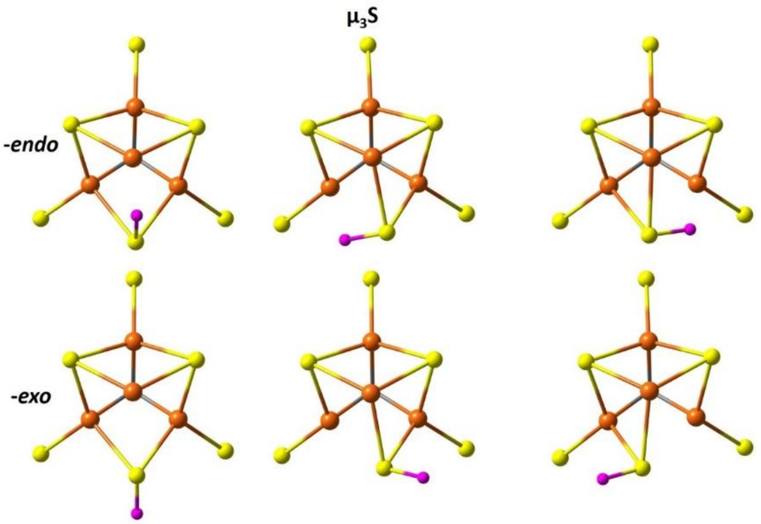

Figure S14. The possible stereochemistry for μ_3-sulfide protonation, shown oriented along the cofactor's approximate C_3-axis.

SVII. Transition State Description

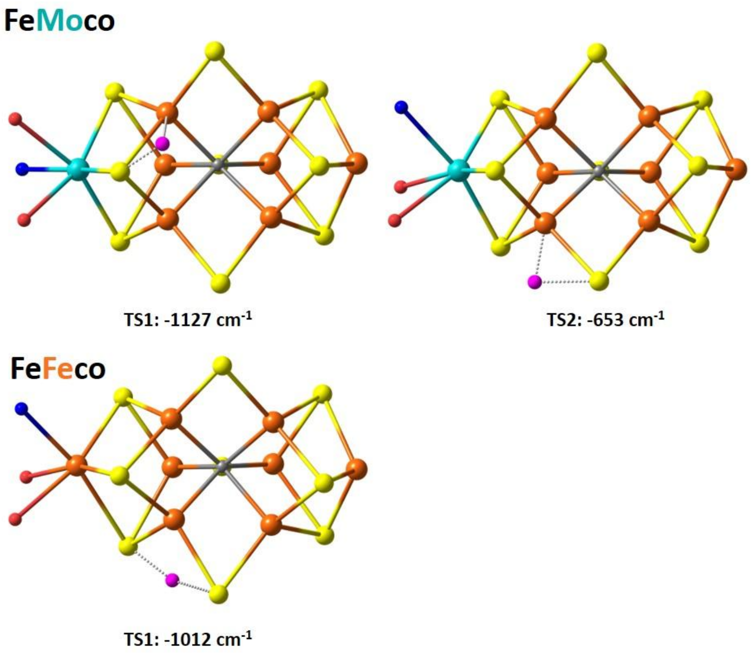

Figure S15. The QM/MM optimized geometries of FeMoco (*top*) and FeFeco's (*bottom*) transition states, connecting their μ_3S3B to μ_2S2B E_1 isomers. The primary coordination sphere and the E_1 state's additional H-center (pink) are highlighted. The transition states' imaginary frequency is provided.

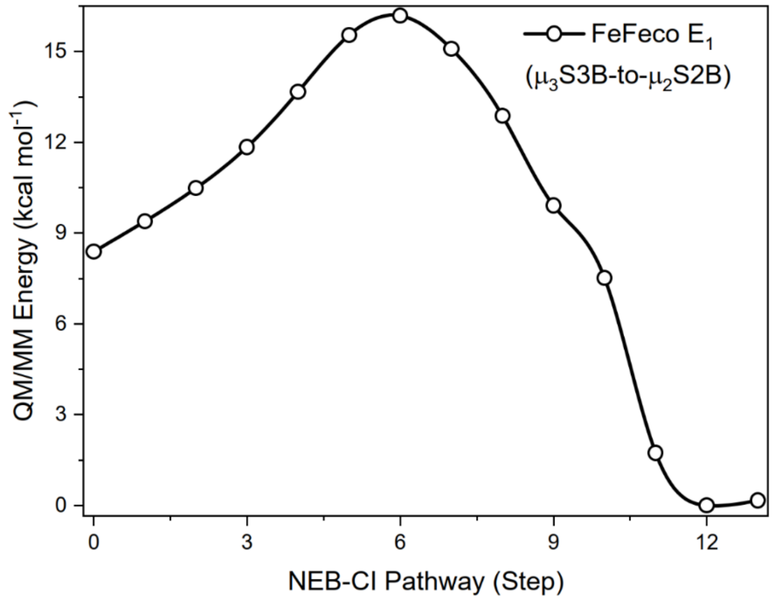

Figure S16. The calculated energy along the NEB-CI pathway, connecting FeFeco's μ_3S3B (Step 0) and μ_2S2B (Step 13) E_1 isomers. The pathway is specific to the BS-2467 solution and the QM/MM energies are relative to the μ_2S2B isomer.

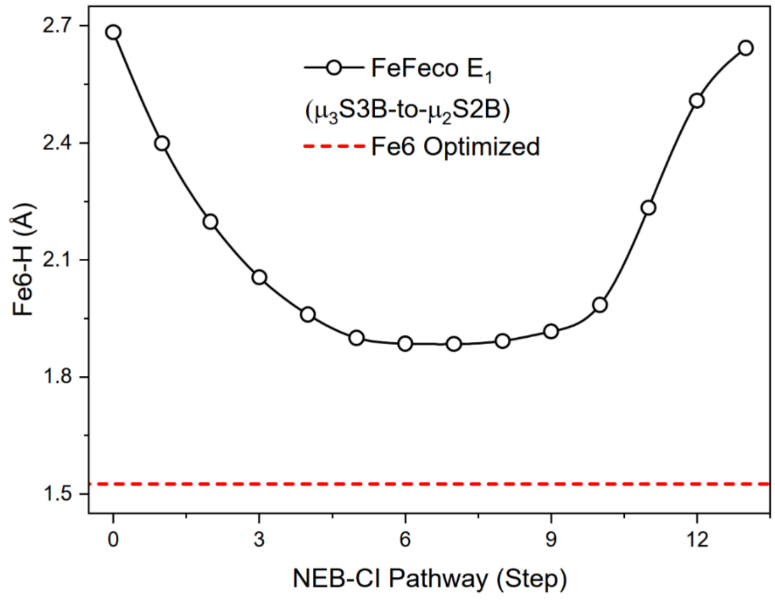

Figure S17. The calculated Fe6-H distance along the NEB-CI pathway, connecting FeFeco's μ_3S3B (Step 0) and μ_2S2B (Step 13) E_1 isomers. The distance of FeFeco's optimized Fe6 E_1 isomer is provided as a reference Fe-hydride bond length.

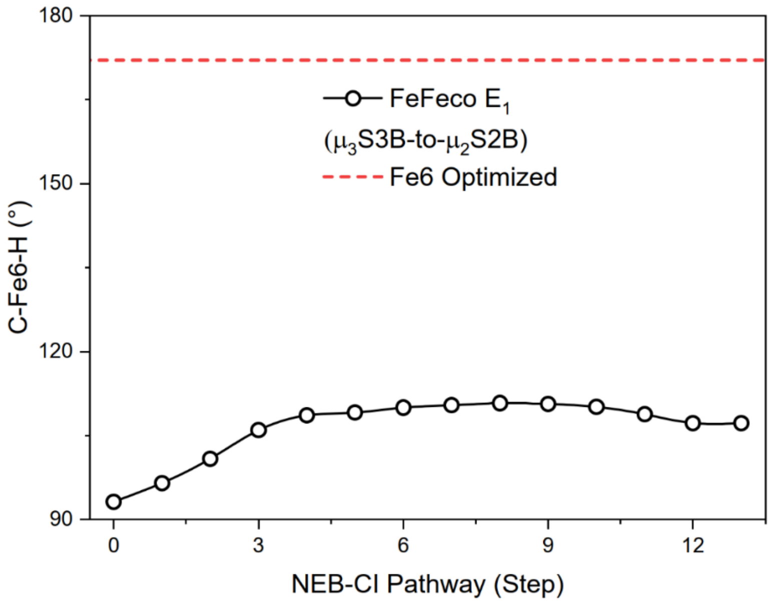

Figure S18. The calculated μ_6C-Fe6-H angle (°) along the NEB-CI pathway, connecting FeFeco's μ_3S3B (Step 0) and μ_2S2B (Step 13) E_1 isomers. The angle of FeFeco's optimized Fe6 E_1 isomer is provided as a reference to the *trans*-conformation of the cluster's Fe-hydrides.

SVIII. Total Electronic Energies

Table S11. The total electronic energy of the QM-(*top*) and MM-component (*bottom*) of the QM(r^2SCAN)/MM optimized E_0 state.

BS-Solution	FeMoco (Eh)	FeFeco (Eh)
BS-235M	-20576.33767, 31658802.84018	-17450.76920, -736.65146
BS-247M	-20576.33670, 31658802.841578	-17450.76765, -736.65088
BS-346M	-20576.33816, 31658802.84088	-17450.76928, -736.64965

Table S12. The total electronic energy of the QM-(*top*) and MM-component (*bottom*) of the QM(r^2SCAN)/MM optimized E_1 isomers of the most stable BS-solution. Select examples calculated by ORCAv5.04 is provided in parenthesis. The BS-solution corresponding to the electronic energy is stated.

E_1 isomer	FeMoco (Eh)	FeFeco (Eh)
μ_2S2B	-20576.90451, 31658802.84264 BS-346M	-17451.33057, -736.65032 (-17451.33062) BS-2467
μ_2S5A	-20576.89698, 31658802.84192 BS-247M	-17451.31853, -736.65532 BS-3467
μ_3S3B	-20576.87634, 31658802.84178 BS-235M	-17451.32458, -736.65073 (-17451.32462)

		BS-235M
μ_3S2A	-20576.88781,<br>31658802.84115<br>BS-235M	-17451.31866,<br>-736.65050<br>BS-235M
Fe6	-20576.88985,<br>31658802.84072<br>BS-346M	-17451.32150,<br>-736.64973<br>BS-346M
Fe7	-20576.8868,<br>31658802.84023<br>BS-247M	-17451.31856,<br>-736.64982<br>BS-247M
Fe2	-20576.89462,<br>31658802.83998<br>BS-235M	-17451.32282,<br>-736.65252<br>BS-235M
Fe3	-20576.89105,<br>31658802.83779<br>BS-235M	-17451.32208,<br>-736.65309<br>BS-235M

Table S13. The total electronic energy of the QM-(*top*) and MM-component (*bottom*) of the QM(TPSSh)/MM optimized E_1 isomers of the most stable BS-solution. The BS-solution corresponding to the electronic energy is provided in parenthesis.

E_1 isomer	FeMoco (Eh)	FeFeco (Eh)
μ_2S2B	-20579.66945,<br>31658802.83833<br>BS-346M	-17454.10413,<br>-736.65293<br>BS-2467
μ_2S5A	-20579.66164,<br>31658802.83790<br>BS-247M	-17454.09217,<br>-736.65832<br>BS-3467
μ_3S3B	-20579.63276,<br>31658802.83649<br>BS-346M	-17454.09941,<br>-736.65459<br>BS-235M
μ_3S2A	-20579.65504,<br>31658802.83756<br>BS-235M	-17454.09334,<br>-736.65394<br>BS-235M
Fe6	-20579.65119,<br>31658802.83620<br>BS-346M	-17454.09298,<br>-736.65237<br>BS-346M
Fe7	-20579.64765,<br>31658802.83603<br>BS-247M	-17454.08929,<br>-736.65264<br>BS-247M
Fe2	-20579.65531,<br>31658802.83545<br>BS-235M	-17454.09442,<br>-736.65597<br>BS-235M
Fe3	-20579.65217,<br>31658802.83314<br>BS-235M	-17454.09386,<br>-736.65705<br>BS-235M

SIX. References

(1) Spatzal, T.; Aksoyoglu, M.; Zhang, L.; Andrade, S. L. A.; Schleicher, E.; Weber, S.; Rees, D. C.; Einsle, O. Evidence for Interstitial Carbon in Nitrogenase FeMo Cofactor. *Science (1979)* **2011**, *334* (6058), 940. DOI: 10.1126/science.1214025.

(2) Trncik, C.; Detemple, F.; Einsle, O. Iron-Only Fe-Nitrogenase Underscores Common Catalytic Principles in Biological Nitrogen Fixation. *Nat Catal* **2023**, *6* (5), 415–424. DOI: 10.1038/s41929-023-00952-1.

(3) Tan, L. L.; Holm, R. H.; Lee, S. C. Structural Analysis of Cubane-Type Iron Clusters. *Polyhedron* **2013**, *58*, 206–217. DOI: 10.1016/j.poly.2013.02.031.

(4) Cao, L.; Caldararu, O.; Ryde, U. Protonation and Reduction of the FeMo Cluster in Nitrogenase Studied by Quantum Mechanics/Molecular Mechanics (QM/MM) Calculations. *J Chem Theory Comput* **2018**, *14* (12), 6653–6678. DOI: 10.1021/acs.jctc.8b00778.

(5) Jiang, H.; Lundgren, K. J. M.; Ryde, U. Protonation of Homocitrate and the E1 State of Fe-Nitrogenase Studied by QM/MM Calculations. *Inorg Chem* **2023**, *62* (48), 19433–19445. DOI: 10.1021/acs.inorgchem.3c02329.

(6) Alwaaly, A.; Dance, I.; Henderson, R. A. Unexpected Explanation for the Enigmatic Acid-Catalysed Reactivity of [Fe4S4X4]2– Clusters. *Chemical Communications* **2014**, *50* (37), 4799–4802. DOI: 10.1039/C4CC00922C.
