# Supplementary figures and images for: Unravelling Catalytic Divergence in Mo- and Fe-Only Nitrogenases: The Role of the Heterometal-Site and Protein Environment from QM/MM Insights
Source: J Am Chem Soc. 2025 Dec 16;147(52):48416–26. doi: 10.1021/jacs.5c20796 (PMC12766731; doi:10.1021/jacs.5c20796)

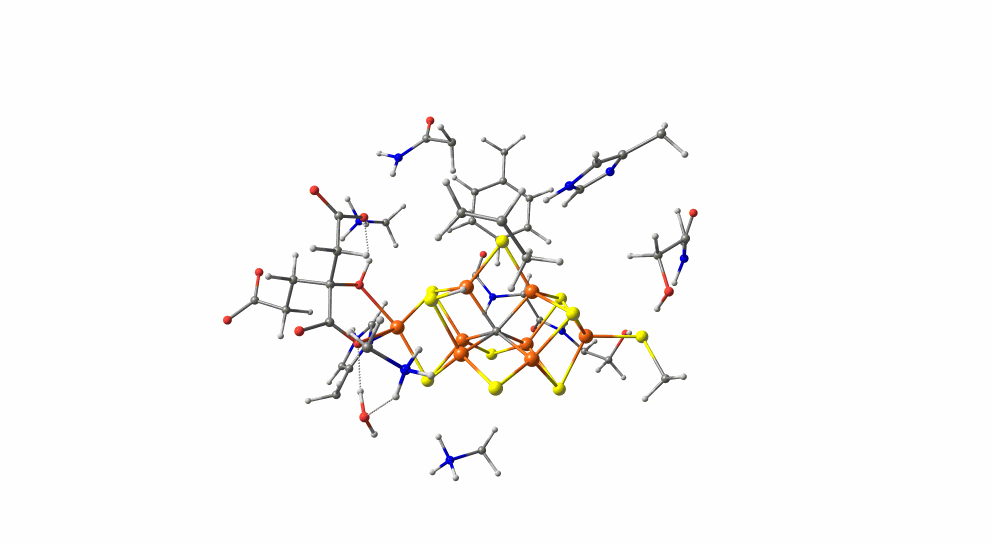

Supplement: Supplementary file 2 [file ja5c20796_si_002.zip › Supplementary/FeFeco/E1/TS1.gif]

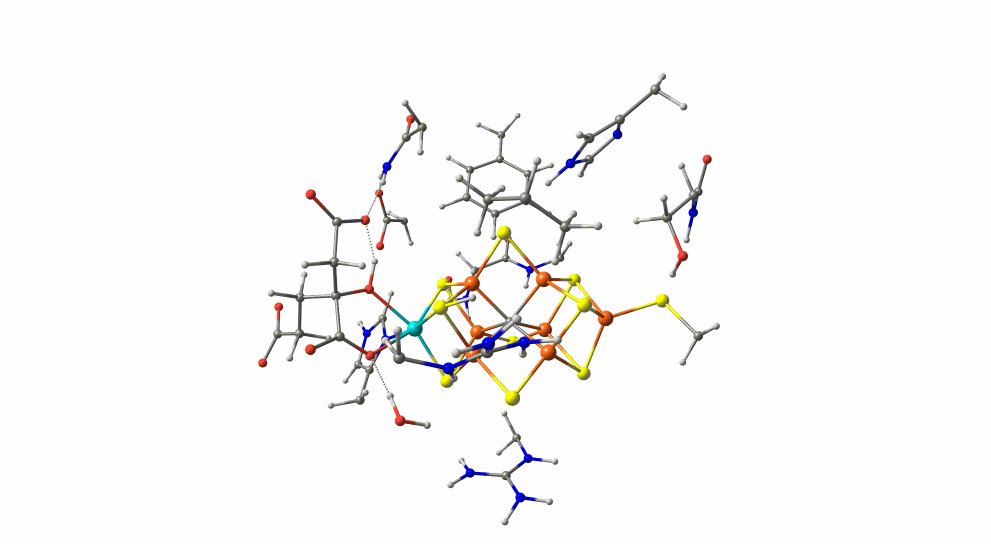

Supplement: Supplementary file 2 [file ja5c20796_si_002.zip › Supplementary/FeMoco/E1/TS1.gif]

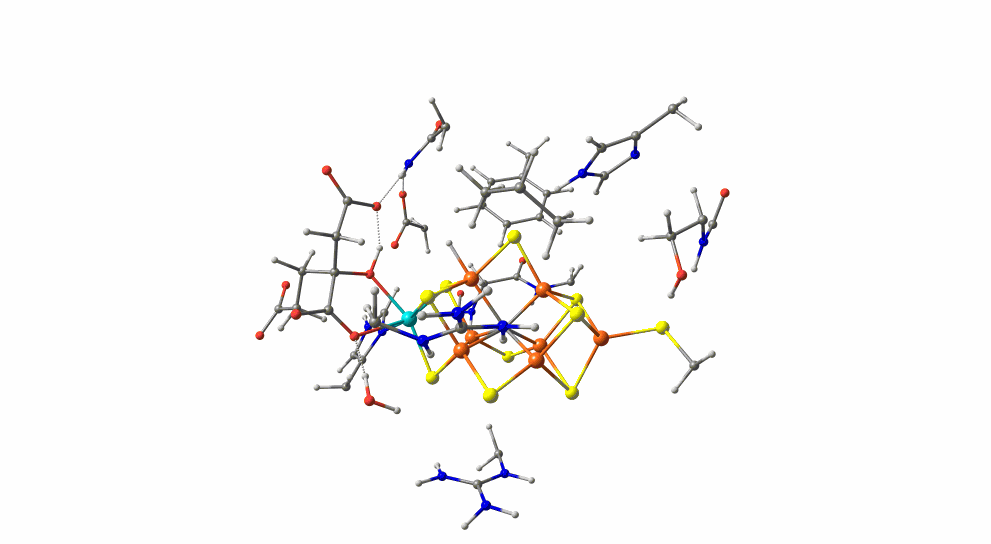

Supplement: Supplementary file 2 [file ja5c20796_si_002.zip › Supplementary/FeMoco/E1/TS2.gif]
